# Cost-effectiveness analysis of direct oral anticoagulants versus low-molecular-weight heparin and no thromboprophylaxis in primary prevention of cancer-associated venous thromboembolism in China

**DOI:** 10.3389/fphar.2024.1373333

**Published:** 2024-09-23

**Authors:** Yue Wu, TianChen Yin, GuiLin Jian, Tao Wan, Benhong Zhou

**Affiliations:** ^1^ Department of Pharmacy, Renmin Hospital, Wuhan University, Wuhan, China; ^2^ School of Pharmaceutical Sciences, Wuhan University, Wuhan, China; ^3^ Department of Pharmacy, Changzhou Jintan District Hospital of Traditional Chinese Medicine, Changzhou, China

**Keywords:** primary thromboprophylaxis, direct oral anticoagulants, cost-effectiveness, cancer-associated venous thromboembolism, low-molecular-weight heparin

## Abstract

**Background and objective:**

Cancer-associated venous thromboembolism (CAVTE) is a preventable, life-threatening complication with a considerable morbidity and mortality. Primary venous thromboembolism (VTE) prophylaxis is currently recommended; however, the health and economic benefits have not been evaluated and compared in China. This study aimed to assess and compare the cost-effectiveness of anticoagulants in primary CAVTE prevention among cancer patients in China.

**Methods:**

A Markov model with a 5-year horizon was established to evaluate the costs and effectiveness of direct oral anticoagulants (DOACs) compared to low-molecular-weight heparins (LMWHs) and no prevention in primary prophylaxis of CAVTE in China. Key clinical outcomes were obtained from the available clinical trials, comparing DOACs (rivaroxaban and apixaban) with LMWHs or with no thromboprophylaxis. Utility and the cost inputs were all obtained from the published literature or local data with public sources. The total costs, quality-adjusted life-years (QALYs), and incremental cost-effectiveness ratios (ICERs) were estimated as the main endpoints of the modal for each strategy. The assessment of uncertainty was performed involving deterministic sensitivity analysis and probabilistic sensitivity analysis (PSA). Impact of time horizon, generic drug price, and individual DOACs were assessed in scenario and subgroup analyses.

**Results:**

Primary prophylaxis using DOACs were projected to yield 1.866 QALYs at a cost of $3,287.893, resulting in the ICERs of $12,895.851 (DOACs vs. no-thromboprophylaxis) and $43,613.184/QALYs (LMWHs vs. DOACs). Sensitivity analysis revealed that ICER was sensitive to the VTE and bleeding risk, drug cost of anticoagulants, self-payment ratio, and overall death rate of cancer. Probabilistic sensitivity analysis showed that DOACs and LMWHs had a 48% and 45% probability of being cost-effective at a 5-year time horizon, respectively. When the time horizon extended to 10 years, DOACs achieved a cost-effective probability of 43%. Among individual DOACs, apixaban was found to be the preferred strategy in VTE prevention due to its incremental health gain with an acceptable cost increase.

**Conclusion:**

Primary thromboprophylaxis with DOACs was cost-effective in cancer patients at a willing-to-pay (WTP) threshold of $37,125.24/QALY in China. Cancer death rate, risk of VTE and major bleeding, and the drug cost assumed greater relevance and importance in the decision-making process for primary thromboprophylaxis in cancer.

## 1 Introduction

Cancer-associated venous thromboembolism (CAVTE) is a prevalent and severe complication observed in the clinical course of malignant tumors (Girardi et al., 2023). In recent years, with the significant improvement in prognosis due to targeted treatment and immunotherapy for tumors, the harm of serious complications on cancer patients has become more prominent (Mulder et al., 2021). The hypercoagulable state, induced by malignance (Ay et al., 2017) and further exacerbated by therapeutic interventions such as chemotherapy, hormonal drugs, or surgical procedures, significantly escalates the incidence of venous thromboembolism (VTE) and causes the overall mortality rate of malignant tumors to increase 2–6 times in the cancer population (Timp et al., 2013). Given the high incidence and mortality rate of CAVTE and its serious impact on patient survival quality, active anticoagulant prophylaxis, especially primary prevention, recently gained prominence worldwide for tumor patients at a high risk of embolism to reduce the occurrence of VTE and improve quality of life (Streiff et al., 2023).

Traditionally, heparin anticoagulants, especially low-molecular-weight heparins (LMWHs), are preferred in VTE prevention due to the controllable anticoagulant strength, the appropriate initiation time, and the low bleeding risk, which is critical for cancer patients (Geerts et al., 2008). Owing to the accumulating safety and efficacy evidence revealed in several large, randomized controlled trials (RCTs) (Rutjes et al., 2020; Baloch et al., 2023), direct oral anticoagulants (DOACs), such as rivaroxaban and apixaban, gradually turned into competition, especially for out-and-ambulatory cancer patients that are referred drugs with good feasibility (Akin et al., 2015). Nevertheless, it still remains unclear whether the clinical benefits offered by DOACs in primary prophylaxis are worth the extra expense, particularly in developing countries like China, which bear a heavier financial burden of cancer than western nations. In this study, we perform a comprehensive analysis to assess the cost-effectiveness of DOACs *versus* LMWHs and no prophylaxis from the perspective of a Chinese payer to provide a reference for the rational drug use of anticoagulants in the cancer population.

## 2 Methods

### 2.1 Model design

**FIGURE 1:**
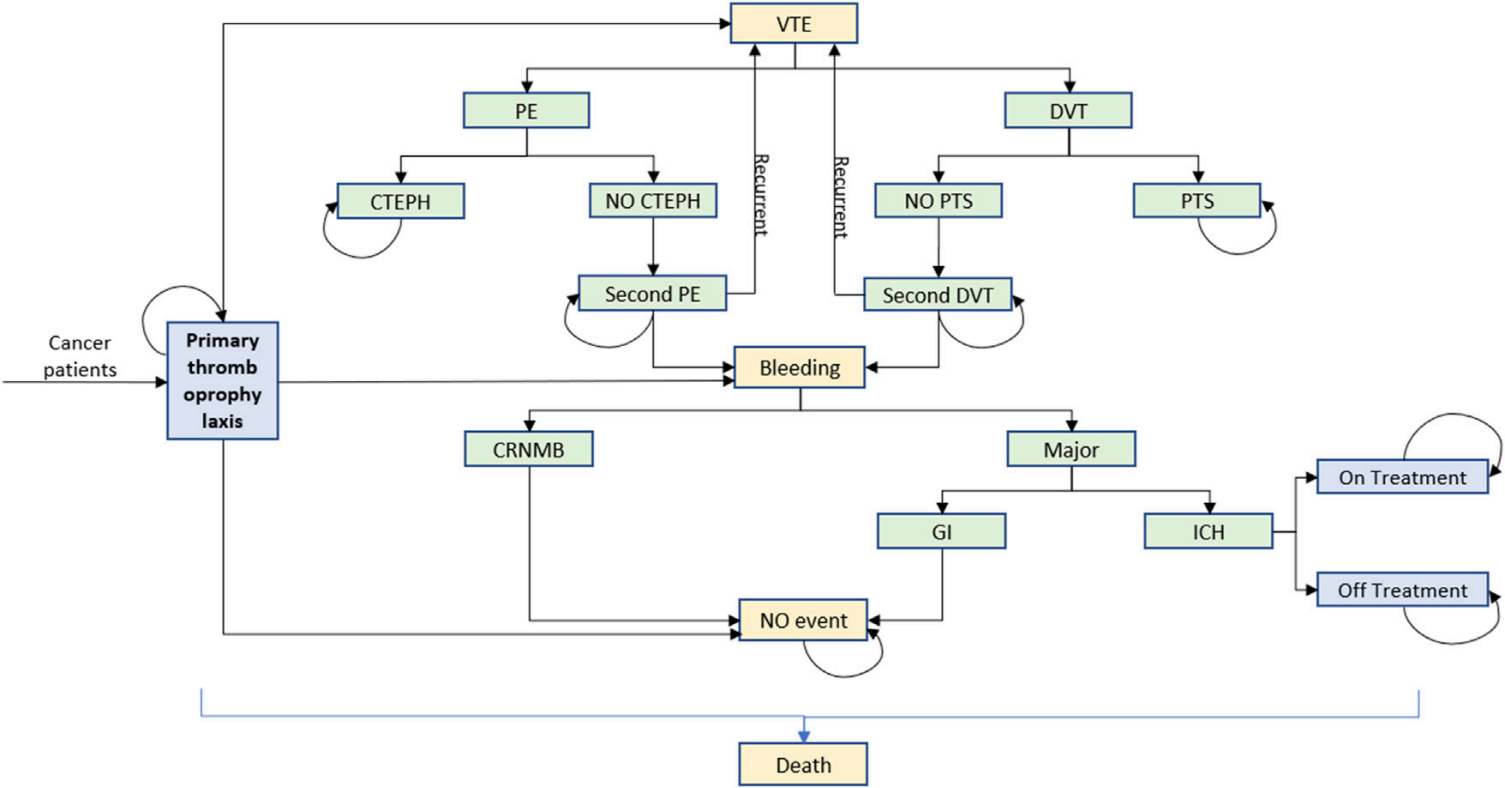
Markov model build with TreeAge Pro 2022. Seven distinct health states were no complications, PE, DVT, ICH, CTEPH, PTS, and death. VTE, venous thromboembolism; PE, pulmonary thromboembolism; DVT, deep vein thrombosis; CTEPH, chronic thromboembolic pulmonary hypertension; PTS, post-thrombotic syndrome; CRNMB, clinically relevant non-major bleeding; GI, gastrointestinal; ICH, intracranial hemorrhage.

We used TreeAge Pro 2022 software to build a Markov model with a cycle period of 1 month and a run period of 5 years based on the clinical and economic impact of cancer survival. According to previously published domestic and international literature (Kimpton et al., 2019; Li et al., 2020), the model was composed of several distinct health states, including no complications, pulmonary embolism (PE), deep vein thrombosis (DVT), intracranial hemorrhage (ICH), chronic thromboembolic pulmonary hypertension (CTEPH), post-thrombotic syndrome (PTS), and death, as shown in Figure 1. The following assumptions were made to reflect the approximate progression of thromboprophylaxis of VTE in patients with cancer, according to the previous literature (Du and Wu, 2020; Li et al., 2020). At the onset of the simulation, all patients were presumed to be in a state free of complications. As each cycle progresses, patients have the potential to either maintain their current health state or transition to a subsequent state due to a clinical event. Recurrence of VTE was allowed in the model in the form of PE or DVT. By a certain chance, CTEPH and PTS would develop after PE and DVT, respectively. A bleeding event was categorized into clinically relevant non-major bleeding (CRNMB) and major bleeding (MB). In our model, MB specifically referred to gastrointestinal hemorrhage (GIB) and ICH as these types of bleeding are associated with significant health loss and substantial clinical resource consumption. A study on the long-term use of antithrombotic treatment in patients with ICH (Ottosen et al., 2016) revealed that approximately 65.7% of patients did not continue with antithrombotic treatment. Consequently, our model simulates a cessation of treatment following an ICH event. In accordance with the clinical guideline of VTE treatment, a transition to therapeutic doses of anticoagulants was also presumed following any occurrence of VTE.

### 2.2 Date and sources

A network meta-analysis was conducted to assess the clinical advantages and bleeding hazards of DOACs and LMWHs in the prevention of CAVTE in comparison to a regimen without thrombotic prophylaxis. Search strategies, study endpoints, and inclusion and exclusion criteria are described in detail in Supplementary Tables S1, S2. A total of 9,024 patients from 22 RCTs were enrolled (Altinbas et al., 2004; Kakkar et al., 2004; Klerk et al., 2005; Wang et al., 2005; Sideras et al., 2006; Agnelli et al., 2009; Perry et al., 2010; van Doormaal et al., 2011; Haas et al., 2012; Levine et al., 2012; Lecumberri et al., 2013; Vadhanraj et al., 2013; Zhang et al., 2013; Zwicker et al., 2013; Zhu et al., 2014; Pelzer et al., 2015; Macbeth et al., 2016; Khorana et al., 2017; Ek et al., 2018; Meyer et al., 2018; Carrier et al., 2019; Khorana et al., 2019). The anticoagulants under investigation in our study included nadroparin, certoparin, dalteparin, enoxaparin, bemiparin, tinzaparin, apixaban, and rivaroxaban (Supplementary Tables S3, S4). The Cochrane bias risk assessment and funnel plots were performed to evaluate the bias among the included RCTs (Supplementary Tables S5–S9). Clinical event rates for patients with no prophylaxis were extracted from published RCTs and combined by a random-effects model (Supplementary Tables S13, S14). The comparative rates of outcomes in the DOACs and LMWHs groups were then calculated by a classic method of applying the corresponding rates of no prophylaxis to the relative risks (RRs) (Supplementary Tables S10, S11) obtained in above meta-analysis. The overall mortality rate was sourced from a 5-year cancer survival survey conducted by the National Health Commission of China (Zeng et al., 2024) and was applied to each group. The long-term death rates of PE, DVT, CTEPH, and PTS were obtained from previous published works (Schulman et al., 2006; Martinez et al., 2018), which are described in detail in Table 1. The transfer probability of recurrent VTE comes from two retrospective studies (Nakano et al., 2021; Ogino et al., 2021). The proportions of major bleeding, ICH, and PE were also extracted and combined from published clinical trials (Kakkar et al., 2004; Klerk et al., 2005; Sideras et al., 2006; Agnelli et al., 2009; Perry et al., 2010; van Doormaal et al., 2011; Haas et al., 2012; Levine et al., 2012; Lecumberri et al., 2013; Vadhanraj et al., 2013; Zhang et al., 2013; Zwicker et al., 2013; Pelzer et al., 2015; Macbeth et al., 2016; Khorana et al., 2017; Ek et al., 2018; Meyer et al., 2018; Carrier et al., 2019; Khorana et al., 2019). In the absence of dynamic data on the occurrence of events, all clinical event rates were converted into monthly probabilities and assumed to remain constant throughout each Markov cycle.

**TABLE 1:** Parameters of imputes, utilities, and costs.

Input variable	Group	Value	Low	Up	SD	Distribution	Source
*Probability or proportion (1 year)*							
Probability of first VTE	DOACs	0.105	0.062	0.178	0.030	Beta	Levine et al. (2012), Carrier et al. (2019), Khorana et al. (2019)
	LMWHs	0.113	0.067	0.191	0.032		Altinbas et al. (2004), Sideras et al. (2006), Agnelli et al. (2009), Perry et al. (2010), van Doormaal et al. (2011), Haas et al. (2012), Lecumberri et al. (2013), Vadhanraj et al. (2013), Zhang et al. (2013), Zwicker et al. (2013), Pelzer et al. (2015), Macbeth et al. (2016), Khorana et al. (2017), Ek et al. (2018), Meyer et al. (2018)
	Placebo	0.194	0.115	0.329	0.055		Altinbas et al. (2004), Sideras et al. (2006), Agnelli et al. (2009), Perry et al. (2010), van Doormaal et al. (2011), Haas et al. (2012), Levine et al. (2012), Lecumberri et al. (2013), Vadhanraj et al. (2013), Zhang et al. (2013), Zwicker et al. (2013), Pelzer et al. (2015), Macbeth et al. (2016), Khorana et al. (2017), Ek et al. (2018), Meyer et al. (2018), Carrier et al. (2019), Khorana et al. (2019)
Probability of bleeding	DOACs	0.144	0.072	0.285	0.055		Levine et al. (2012), Carrier et al. (2019), Khorana et al. (2019)
	LMWHs	0.154	0.077	0.306	0.058		Kakkar et al. (2004), Klerk et al. (2005), Agnelli et al. (2009), van Doormaal et al. (2011), Haas et al. (2012), Lecumberri et al. (2013), Zhang et al. (2013), Macbeth et al. (2016), Khorana et al. (2017)
	Placebo	0.092	0.046	0.183	0.035		Kakkar et al. (2004), Klerk et al. (2005), Agnelli et al. (2009), van Doormaal et al. (2011), Haas et al. (2012), Levine et al. (2012), Lecumberri et al. (2013), Zhang et al. (2013), Macbeth et al. (2016), Khorana et al. (2017), Carrier et al. (2019), Khorana et al. (2019)
First VTE is a PE (%)	DOACs	0.370	0.333	0.407	0.019		Levine et al. (2012), Carrier et al. (2019), Khorana et al. (2019)
	LMWHs	0.441	0.397	0.485	0.023		Wang et al. (2005), Agnelli et al. (2009), Perry et al. (2010), van Doormaal et al. (2011), Haas et al. (2012), Vadhanraj et al. (2013), Zhang et al. (2013), Pelzer et al. (2015), Macbeth et al. (2016), Khorana et al. (2017), Ek et al. (2018)
	Placebo	0.456	0.410	0.501	0.023		Wang et al. (2005), Agnelli et al. (2009), Perry et al. (2010), van Doormaal et al. (2011), Haas et al. (2012), Levine et al. (2012), Vadhanraj et al. (2013), Zhang et al. (2013), Pelzer et al. (2015), Macbeth et al. (2016), Khorana et al. (2017), Ek et al. (2018), Carrier et al. (2019), Khorana et al. (2019)
Proportion of major bleeding (%)	DOACs	0.357	0.321	0.393	0.018		Levine et al. (2012), Carrier et al. (2019), Khorana et al. (2019)
	LMWHs	0.236	0.212	0.259	0.012		Kakkar et al. (2004), Klerk et al. (2005), Agnelli et al. (2009), van Doormaal et al. (2011), Haas et al. (2012), Lecumberri et al. (2013), Zhang et al. (2013), Macbeth et al. (2016), Khorana et al. (2017)
	Placebo	0.285	0.256	0.313	0.015		Kakkar et al. (2004), Klerk et al. (2005), Agnelli et al. (2009), van Doormaal et al. (2011), Haas et al. (2012), Levine et al. (2012), Lecumberri et al. (2013), Zhang et al. (2013), Macbeth et al. (2016), Khorana et al. (2017), Carrier et al. (2019), Khorana et al. (2019)
ICH in major bleeding (%)	DOACs	0.125	0.113	0.138	0.006		Kim et al. (2018)
	LMWHs	0.229	0.206	0.252	0.012		Büller et al. (2012)
	Placebo	0.231	0.208	0.254	0.012		Fox et al. (2011)
Proportion of anticoagulant termination after ICH (%)	-	0.657	0.591	0.722	0.034		Ottosen et al. (2016)
Annual death rate of cancer patients	-	0.153	0.132	0.176	0.011		Zeng et al. (2024)
Recurrent VTE after DVT	-	0.027	0.024	0.030	0.002		Ogino et al. (2021)
Bleeding after DVT	-	0.054	0.048	0.060	0.003		Ogino et al. (2021)
Death in DVT	-	0.316	0.278	0.357	0.020		Ogino et al. (2021)
Recurrent VTE after PE	-	0.057	0.052	0.063	0.003		Nakano et al. (2021)
Bleeding in PE	-	0.069	0.062	0.076	0.004		Nakano et al. (2021)
Death in PE	-	0.402	0.362	0.443	0.021		Nakano et al. (2021)
Recurrent VTE after ICH	-	0.035	0.032	0.039	0.002		Ottosen et al. (2016)
Bleeding in treated ICH		0.079	0.071	0.087	0.004		Ottosen et al. (2016)
Bleeding in off-treated ICH		0.086	0.077	0.0946	0.004		Ottosen et al. (2016)
Death in on-treatment ICH	-	0.097	0.069	0.137	0.017		Nielsen et al. (2015)
Death in off-treatment ICH	-	0.191	0.016	0.226	0.054		Nielsen et al. (2015)
Probability of death in PTS	-	0.033	0.030	0.037	0.002		Schulman et al. (2006)
*Probability of PTS in treated DVT*							
Year 1	-	0.180	0.109	0.251	0.036	Beta	Du and Wu (2020)
Year 2	-	0.079	0.048	0.110	0.016		
Years 3–5	-	0.023	0.014	0.032	0.005		
*Probability of CTEPH in treated PE*							
Year 1	-	0.031	0.019	0.043	0.006	Beta	Du and Wu (2020)
Year 2	-	0.007	0.004	0.010	0.002		
*Probability of death in CTEPH*							
1–90 days	-	0.327	0.202	0.499	0.076	Beta	Martinez et al. (2018)
91–365 days	-	0.175	0.114	0.256	0.036		
Year 2	-	0.110	0.060	0.184	0.032		
Year 3	-	0.081	0.048	0.129	0.021		
*Probability or proportion (1 year) of subgroup analysis*							
Probability of first VTE	Apixaban	0.072	0.043	0.122	0.020	Beta	Levine et al. (2012), Carrier et al. (2019)
	Rivaroxaban	0.132	0.078	0.224	0.037		Khorana et al. (2019)
Probability of bleeding	Apixaban	0.143	0.071	0.284	0.054		Levine et al. (2012), Carrier et al. (2019)
	Rivaroxaban	0.145	0.073	0.289	0.055		Khorana et al. (2019)
First VTE is a PE (%)	Apixaban	0.417	0.375	0.458	0.021		Levine et al. (2012), Carrier et al. (2019)
	Rivaroxaban	0.333	0.300	0.367	0.017		Khorana et al. (2019)
Proportion of major bleeding (%)	Apixaban	0.324	0.292	0.357	0.017		Levine et al. (2012), Carrier et al. (2019)
	Rivaroxaban	0.421	0.379	0.463	0.022		Khorana et al. (2019)
*Utility*							
ICH		0.330	0.260	0.400	0.036	Beta	Wumaier et al. (2021)
Cancer without VTE		0.650	0.616	0.672	0.014		Du and Wu (2020)
DVT		0.610	0.514	0.678	0.042		Du and Wu (2020)
PE		0.620	0.477	0.725	0.063		Du and Wu (2020)
PTS		0.500	0.320	0.650	0.084		Du and Wu (2020)
CTEPH		0.630	0.520	0.730	0.054		Du and Wu (2020)
Death		0	-	-			-
*Disutility*							
DVT		0.190	0.060	0.450	0.010	Beta	Li et al. (2020)
PE		0.250	0.090	0.550	0.117		Li et al. (2020)
Major bleeding		0.270	0.246	0.294	0.012		Kimpton et al. (2019)
CRNMB		0.013	0.010	0.016	0.002		Kimpton et al. (2019)
ICH		0.470	0.340	0.600	0.066		Kimpton et al. (2019)
PTS		0.050	0.028	0.072	0.011		Kimpton et al. (2019)
CTEPH		0.360	0.344	0.376	0.008		Kimpton et al. (2019)
*Cost of drug prevention (one-cycle)*							
DOACs		160.372	100.144	275.423	0.258	Log-normal	Public database (yaozh.com)
LMWHs		348.113	264.463	440.683	0.130		
Apixaban		222.621	144.082	364.422	0.237		
Rivaroxaban		98.122	56.205	186.424	0.306		
Original drugs	DOACs	263.927	179.172	384.985	0.195		
	LMWHs	350.800	277.870	440.683	0.118		
Generic drugs	DOACs	91.697	46.067	133.967	0.272		
	LMWHs	327.801	219.037	434.677	0.175		
*Cost of drug treatment (3 months)*							
DOACs		1,048.517	654.152	1,802.819	0.259	Log-normal	Public database (yaozh.com)
LMWHs		1,547.152	1,130.174	2,131.714	0.162		
Apixaban		1,439.619	931.732	2,356.597	0.237		
Rivaroxaban		657.415	376.572	1,249.041	0.036		
Original drugs	DOACs	1,730.316	1,165.273	2,537.349	0.199		
	LMWHs	1,590.548	1,114.712	2,131.714	0.165		
Generic drugs	DOACs	599.515	300.389	877.713	0.274		
	LMWHs	1,447.068	900.384	1,996.903	0.203		
*Cost of events (one-time)*							
DVT		693.000	329.000	941.000	0.268	Log-normal	Du and Wu (2020)
PE		1,121.000	448.000	1,793.000	0.354		Du and Wu (2020)
ICH		4,378.347	2,677.667	6,066.547	0.209		China Health Statistical Yearbook 2022
GI bleeding		1,876.013	978.700	3,209.564	0.303		China Health Statistical Yearbook 2022
CRNMB		8.250	5.770	10.720	0.158		Yang and Wu (2020)
Post-ICH (1 year)		2,527.000	2,269.143	2,784.857	0.052		China Health Statistical Yearbook 2022
PTS (1 year)		1,872.904	1,498.323	2,247.490	0.103		Chen et al. (2011)
CTEPH (1 year)		10,747.988	8,598.390	12,897.586	0.103		Chen et al. (2011)

### 2.3 Costs and utility inputs

The cost of this study is aligned with the current Chinese healthcare system. From the perspective of patients, we considered only the direct medical costs, including the costs of drug, expenses related to the management of clinical events, and local self-copay ratio in Chinese Medicare. All costs are expressed in United States dollars, using the average exchange rate of 2023 (¥ = $0.144). The cost of the drug is calculated by multiplying the unit price by the dosage. The unit price of drugs was obtained from the average price listed in the public database (yaozh.com). The dosage for anticoagulant prevention or treatment was incorporated in the model in consistence with the NCCN guidelines (Streiff et al., 2023) or the drug package insert. In accordance with the guidelines for the management of cancer-associated thrombosis, it was assumed that prophylactic medication would be administered lifelong, while three-month therapeutic doses were supposed in symptomatic DVT and PE before transitioning to prophylactic dosing. The treatment costs of ICH and gastrointestinal bleeding were both derived from “China Health Statistical Yearbook 2022.” Patients with PTS and CTEPH require long-term treatment, and the annual cost is based on clinical data from six hospitals in China (Chen et al., 2011).

Utility value is a widely used parameter for assessing the impact of an intervention on the quality of life. For cancer patients without complications, a baseline utility of 0.650 was adopted in accordance with the previous literature (Du and Wu, 2020). The permanent dis-utilities of 0.250, 0.190, 0.470, 0.360, and 0.050 were used for PE, DVT, ICH, CTEPH, and PTS, respectively, according to the previous literature studies (Table 1) to calculate the long-term impact of these events on health. One-time dis-utilities of 0.270 and 0.013 were assigned for BIG and CRNMB due to their transient impact on health (Table 1). All costs and utilities were discounted at an annual rate of 5%, according to the recommendation of China Guidelines for pharmacoeconomic evaluations.

## 3 Analyses

The key metrics assessed in base-case analysis included incremental costs, incremental quality-adjusted life-years (QALYs), and the incremental cost-effectiveness ratio (ICER). In light of the current lack of a recommended willingness-to-pay threshold (WTP) in China, we employed three times the *per capita* gross national product (GDP) of 2022, which amounts to $37,125.240, as a reference point for assessing the cost-effectiveness of various treatment options. To explore the influence of parameter uncertainty on the final results, scenario analysis, one-way sensitivity analysis, and probabilistic sensitivity analysis (PSA) were also performed in this study. In one-way sensitivity analysis, the parameter inputs were assumed to vary over their 95% confidence intervals. If a confidence interval was not provided, a variation of ±20% from the mean values was estimated for the costs and ±10% for the transfer probabilities. In the scenario analysis, we explored variations in time horizon and the cost reduction associated with the introduction of generic drugs after patent expiry. In PSA, appropriate distribution functions were assigned to each parameter according to the type of data. Beta distributions were applied for clinical outcomes and health utilities, while log-normal distributions were used for the costs. Relative risks of clinical events in DOACs and LMWHs, in comparison to no prophylaxis, were assigned to beta distributions. We conducted a 10,000-subject Monte Carlo simulation based on these variable distributions, allowing all parameter inputs to vary stochastically in the PSA. The PSA results are visually presented as scatterplots.

## 4 Results

### 4.1 Base-case analysis

In base-case analysis, with a 5-year projected time, the estimated outcomes for primary prophylaxis using DOACs were projected to yield 1.866 QALYs at a cost of $ 3,287.893. In comparison, prophylaxis with LMWHs resulted in 1.915 QALYs at a cost of $ 5,424.939, while opting for no thromboprophylaxis achieved 1.779 QALYs at a cost of $2,165.954. Compared with no prophylaxis, DOACs and LMWHs were associated with a gain of 0.087 and 0.136 QALYs at additional costs of $ 1,121.939 and $3,258.985, respectively. The ICERs were $12,895.851 and 23,963.125 per QALY, respectively (Table 2). These ICERs were less than WTP, indicating that primary thrombosis prophylaxis was cost-effective in the prevention of CAVTE in the cancer population. When DOACs were set as a competitor drug, prophylaxis with LMWHs was associated with a gain of 0.049 QALYs at the incremental cost of $2,137.046. The estimated ICER was $43,613.184/QALY. This value exceeded three times the *per capita* GDP of China, indicating that DOACs were the preferable anticoagulants over the traditional LMWHs for VTE primary prophylaxis in malignancy.

**TABLE 2:** Cost-effectiveness analysis.

Time horizon	Treatment therapies in the order of cost			Placebo as the common reference	LMWHs as the common reference
	Treatment strategy	Cost ($)	ICER ($/QALY)	ICER ($/QALY)	ICER ($/QALY)
3 years	Placebo	1,327.800	1.335	-	-
	DOACs	2,211.324	1.376	21,549.366	56,664.926
	LMWHs	3,741.277	1.403	35,492.309	-
	Apixaban	2,491.005	1.391	20,771.518	104,189.333
	Rivaroxaban	1,924.718	1.365	19,897.267	47,804.184
5 years	Placebo	2,165.954	1.779	-	-
	DOACs	3,287.893	1.866	12,895.851	43,613.184
	LMWHs	5,424.939	1.915	23,963.125	-
	Apixaban	3,611.529	1.893	12,680.482	82,427.727
	Rivaroxaban	2,943.836	1.845	11,786.091	35,444.329
10 years	Placebo	3,666.882	2.278	-	-
	DOACs	5,010.023	2.428	8,954.273	34,843.880
	LMWHs	7,902.065	2.511	18,176.751	-
	Apixaban	5,266.992	2.470	8,333.906	64,270.073
	Rivaroxaban	4,688.537	2.403	8,173.240	29,754.889

In addition, the QALYs and the costs of individual DOACs, including apixaban and rivaroxaban, were also calculated. Compared with no prophylaxis, thromboprophylaxis with either apixaban or rivaroxaban resulted in higher overall costs and improved health outcomes. The predicted costs were $3,611.529 for apixaban and $2,943.836 for rivaroxaban, while the health gained were 1.893 QALYs and 1.845 QALYs, respectively. The ICERs were estimated to be $13910.27/QALY when apixaban was compared to rivaroxaban, suggesting that apixaban was a more cost-effective drug for thromboprophylaxis (Table 2).

### 4.2 Scenario analysis

In the scenario analysis, the impacts of the time horizons and market access of generic drugs after patent expiry were examined. With the extension of the time horizon, incremental QALYs and treatment costs were estimated but with a gradual decline in ICER in DOACs compared with no prophylaxis, which was from $21,549.366/QALY for a 3-year period to $8,954.273/QALY for a 10-year period. This suggested that long-term primary prophylaxis with anticoagulants leads to improved benefits in health and economic aspects (Table 2). When compared with LMWHs, DOACs exhibited decreased ICERs in 10-year simulation ($43,613.184//QALY vs. $34,843.880//QALY) and shifted from a dominating to dominated status. This finding might be related to the relative higher death risk in DOACs, leading to the lower health gain over a longer time simulation. When original anticoagulants and their generic counterparts were incorporated, it was observed that prophylaxis with both original and generic anticoagulants were cost-effective. However, generic DOACs produced the lowest ICER, falling below the GDP *per capita* threshold (Table 3). It suggested that generic DOACs were the preferred prophylaxis option for VTE prevention in China.

**TABLE 3:** Cost-effectiveness analysis of original and generic drugs.

Time horizon	Treatment therapies in the order of cost			Placebo as the common reference	DOACs (generic drugs) as the common reference
	Treatment strategy	Cost ($)	QALY	ICER ($/QALY)	ICER ($/QALY)
3 years	Placebo	1,327.800	1.335	-	-
	DOACs (original drugs)	3,036.215	1.376	41,668.659	-
	DOACs (generic drugs)	1 ,664.458	1.376	8,211.171	-
	LMWHs (original drugs)	3,764.659	1.403	35,836.162	77,785.222
	LMWHs (generic drugs)	3,577.227	1.403	33,079.809	70,843.296
5 years	Placebo	2,165.954	1.779	-	-
	DOACs (original drugs)	4,422.729	1.866	25,939.943	-
	DOACs (generic drugs)	2,535.596	1.866	4,248.759	-
	LMWHs (original drugs)	5,457.939	1.915	24,205.772	59,639.653
	LMWHs (generic drugs)	5,198.114	1.915	22,295.294	54,337.102
10 years	Placebo	3,666.882	2.278	-	-
	DOACs (original drugs)	6,517.338	2.428	19,003.04	-
	DOACs (generic drugs)	4,010.838	2.428	2,293.04	-
	LMWHs (original drugs)	7,946.687	2.511	18,368.262	47,419.867
	LMWHs (generic drugs)	7,598.873	2.511	16,875.498	43,229.337

### 4.3 Sensitivity analyses

**FIGURE 2:**
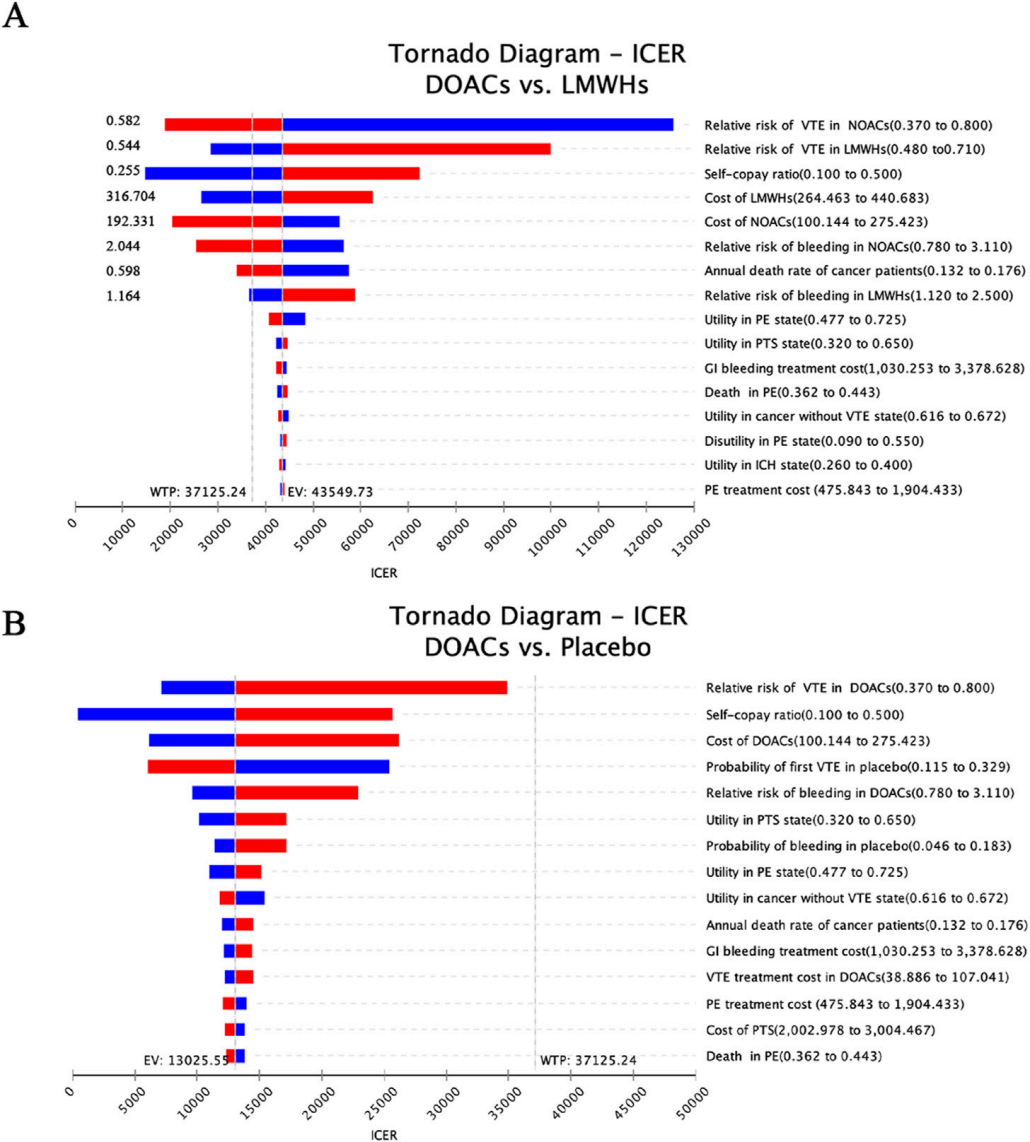
Tornado diagram illustrating the results of the one-way sensitivity analysis **(A)** DOACs vs LMWHs and **(B)** DOACs vs Placebo. Each bar represents the range of variation within the set intervals, ordered from top to bottom by the magnitude of their impact.

Figure 2 displays the univariate sensitivity analyses of the individual parameter inputs that exerted the greatest influence on the ICERs, arranged according to their respective levels of impact. When DOACs were compared with no prophylaxis, the relative risk of VTE in DOACs was found to have a great impact on the ICER, followed by the proportion of drug reimbursement and the cost of DOACs. Furthermore, it is observed that changing all the inputs within their reasonable range only resulted in the changes in ICER values but not in the reversion of the final result, indicating that DOACs are robustly cost-effective compared to no prophylaxis (Figure 2A).

When LMWHs were employed as counterparts to DOACs, it was discovered that the result is sensitive to several factors: the VTE risks, self-copay ratio, cost of DOACs and LMWHs, bleeding risks of thromboprophylaxis, and the annual death rate of cancer (Figure 2B). Specifically, when the VTE risk reached 0.582, the bleeding risk exceeded 2.044, or the drug cost surpassed $192.331, the ICERs for DOACs fell below the willingness-to-pay threshold of $37,125.24. This indicated a shift in strategy for DOACs from being dominant to being dominated. Conversely, LMWHs demonstrated a cost-effective advantage over DOACs in VTE prevention in cancer patients when a low risk of VTE and bleeding (less than 0.544 and 1.164, respectively) or a price decrease (lower than $316.704) was applied. Moreover, an increased self-copay ratio and a smaller death rate were found to enhance the cost-effective advantage of DOACs in the primary prophylaxis of CAVTE.

**FIGURE 3:**
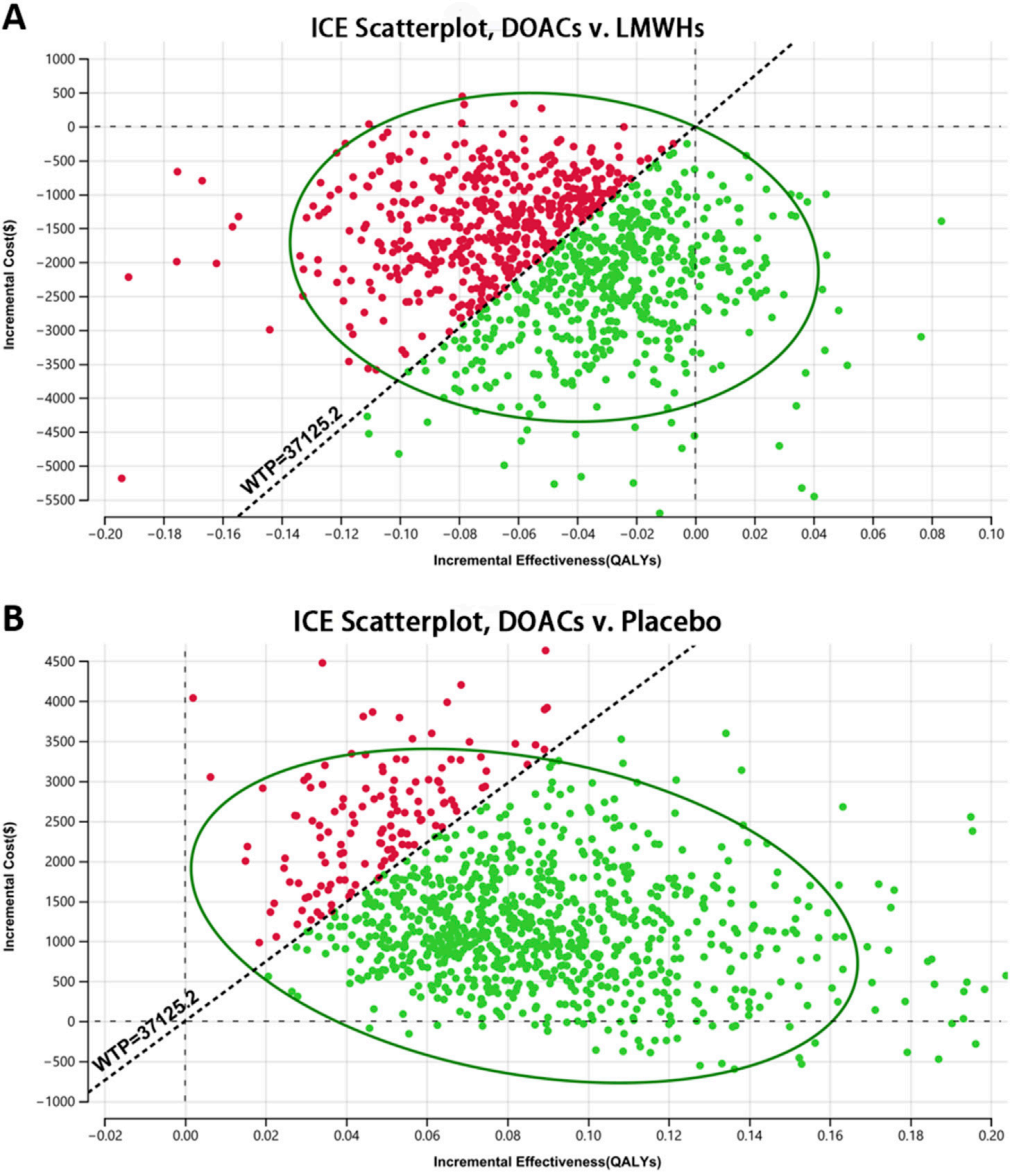
Scatter plot of the probabilistic sensitivity analysis (PSA) **(A)** DOACs vs. LMWHs and **(B)** DOACs vs. placebo. Scatter plot diagram illustrated the results of a 10,000-subject Monte Carlo simulation, estimating the probability of acceptance for each treatment strategy.

**FIGURE 4:**
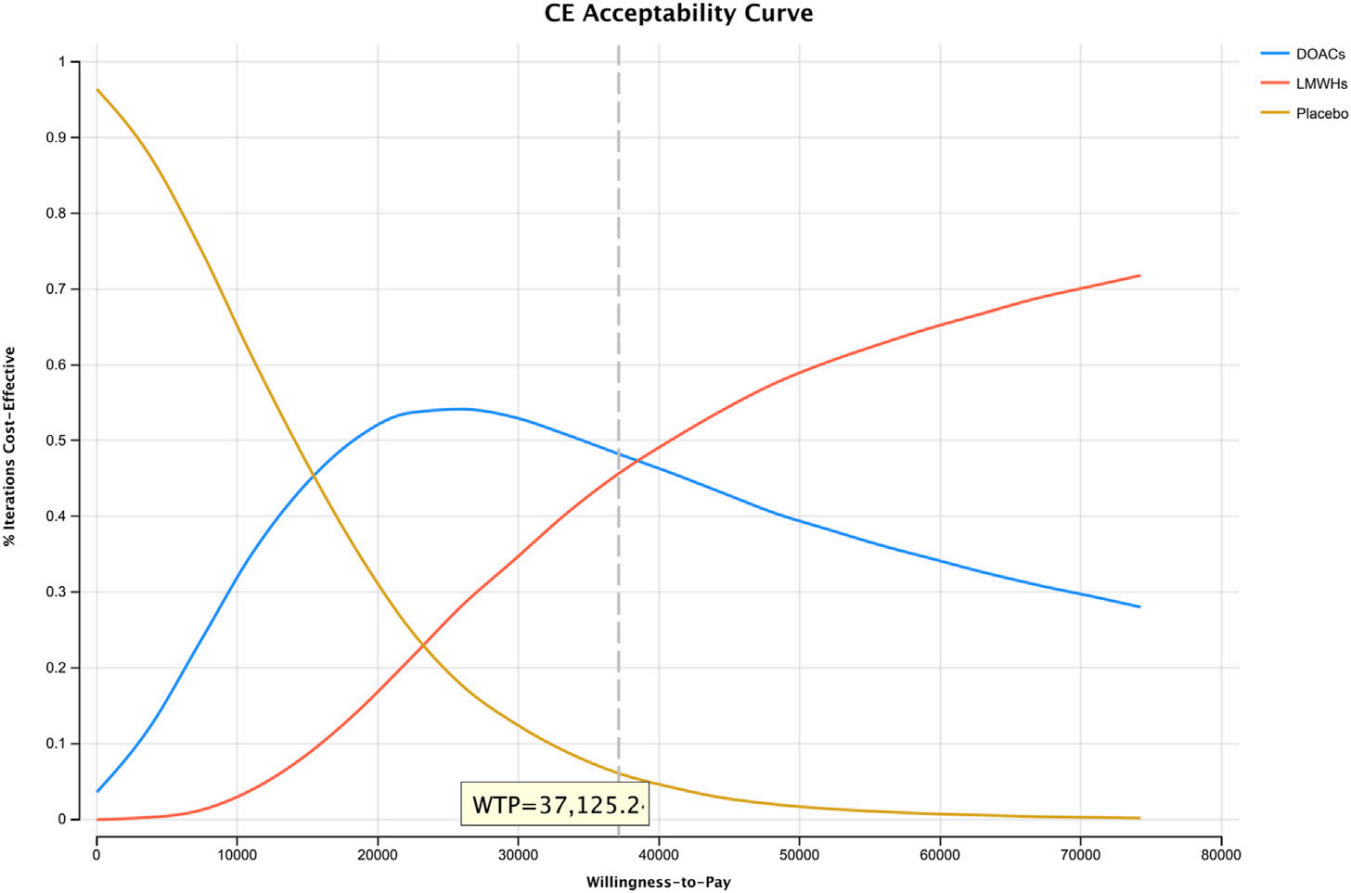
Willingness-to-pay curve estimated from a 10,000-subject Monte Carlo simulation representing the change in the probability of acceptance for three drugs as the WTP increases.

Figure 3 represents the results of PSA of DOACs, LMWHs, and no prophylaxis over a time horizon of 5-year, and detailed results are listed in Table 4. Primary prophylaxis with DOACs and LMWHs resulted in the average costs of $ 3,352.552 and $ 5,313.533, respectively, and the total cost of no prophylaxis was estimated to be $ 2,066.495. Meanwhile, the corresponding health gain was 1.865, 1.915, and 1.781 QALY, respectively. A cost-effectiveness acceptability curve was plotted to illustrate the proportion of simulations that were cost-effective at WTP values (Figure 4). Using a WTP threshold of $37,125.240, the probability of acceptance was 48% for DOACs, 45% for LMWHs, and 7% for no prophylaxis. When a 10-year time horizon was applied, these probabilities changed into 43% and 55% (Supplementary Material). For individual DOACs, the average costs, QALYs, and ICERs were also calculated in PSA. Although minimal additional costs and health gain were required, apixaban was found to be more cost-effective than rivaroxaban. These findings were closely aligned with those of the base-case analysis. Acceptable probabilities of apixaban were estimated to be 66% and 68% for the 5-year and 10-year time periods, while rivaroxaban had acceptable probabilities of 29% and 31%, respectively (Supplementary Tables S15, S16).

**TABLE 4:** Probabilistic sensitivity analysis.

Time horizon	Treatment therapies in the order of cost			Placebo as the common reference	LMWHs as the common reference
	Treatment strategy	Cost ($)	ICER ($/QALY)	ICER ($/QALY)	ICER ($/QALY)
3 years	Placebo	1,255.922	1.334		-
	DOACs	2,255.532	1.375	24,380.732	51,612.370
	LMWHs	3,649.066	1.402	35,193.294	-
	Apixaban	2,560.224	1.390	23,291.107	90,736.833
	Rivaroxaban	1,943.563	1.364	22,921.367	44,881.658
5 years	Placebo	2,066.495	1.781		-
	DOACs	3,352.552	1.865	15,310.202	39,219.620
	LMWHs	5,313.533	1.915	24,231.627	-
	Apixaban	3,699.536	1.893	14,580.723	73,363.500
	Rivaroxaban	2,946.522	1.847	13,333.742	34,808.985
10 years	Placebo	3,524.153	2.282		-
	DOACs	5,071.229	2.430	10,453.216	31,441.871
	LMWHs	7,743.788	2.515	18,110.021	-
	Apixaban	5,398.561	2.475	9,711.9585	58,630.675
	Rivaroxaban	4,685.006	2.411	8,998.8605	29,411.365

## 5 Discussion

VTE is a burdensome but preventable complication that frequently occurs in patients with active cancer. Given the dramatic improvement of cancer survival from targeted treatment and immunotherapy, the health and potential economic benefits of preventing serious complications have become increasingly important in the management of cancer. In this research, we focused on the primary thromboprophylaxis of VTE in cancer patients to perform a cost-effectiveness analysis comparing DOACs with traditional LMWHs and no prophylaxis comprehensively. This is the first study to achieve an indirect comparison of DOACs with a network meta-analysis approach to assess their cost-effectiveness in primary prevention based on medical costs and resources used in healthcare decisions in China.

The administration of anticoagulant prophylaxis in cancer patients is a complex issue due to the increased bleeding risk associated with tumors. For a long time, only the prevention of recurrent VTE was emphasized (de Jong et al., 2020), and the available anticoagulant drugs was limited to low-molecular-weight heparin, which has a relatively controllable risk of bleeding (Streiff et al., 2018). There is a scarcity of research on the economic evaluation of primary VTE prevention in cancer patients, particularly when comparing different types of anticoagulants. In this research, incorporating the latest RCT results and the evidence of a comprehensive systematic literature review, thromboprophylaxis with DOACs was studied in cancer patients compared with LMWHs from a pharmacoeconomic perspective. The results revealed that DOACs offered clinical benefits over those of LMWHs but at a lower cost. The estimated ICER was $43,613.184/QALYs (LMWHs vs. DOACs), indicating that DOACs are a more cost-effective option for primary VTE prevention in cancer patients in the current Chinese social environment. This result is consistent with several previous works (Lanitis et al., 2016; Heisen et al., 2017; Shin et al., 2022) that compared DOACs with LMWHs in the secondary prevention of cancer-associated thrombosis in the US healthcare system. However, another study conducted in China suggested that the cost-effectiveness of DOACs for thromboprophylaxis in patients initiating chemotherapy is unlikely (Du and Wu, 2020). The inconsistency in conclusions may arise from various factors. First, both the previous report and our research identified the price of DOACs as one of the most significant parameter inputs that have a substantial impact on the result. In our research, we applied lower prices after the patent expired in accordance with the marketing entry of generic drugs in China. Despite a slight increase in bleeding and additional costs compared to no-thrombosis prophylaxis, DOACs ultimately demonstrated their current pharmacoeconomic advantage in VTE prevention in China. Second, our model considered detailed bleeding events, such as ICH, GIB, and CRNMB. This approach aims to reflect the natural process and incorporate the best available evidence regarding the performance of thromboprophylaxis in patients with cancer. Additionally, a comprehensive analysis of 22 randomized controlled trails, including DOACs, LMWHs, and no thromboprophylaxis, was conducted in this work to assess the clinical benefits of thromboprophylaxis in the prevention of cancer-associated VTE, while evidence of LMWHs was excluded in the previous paper. We think that all the above changes might lead to a shift toward DOACs becoming the dominating strategy.

Clinical practice guidelines and data from numerous clinical trials have established that appropriate VTE prophylaxis is both safe and effective. However, practice surveys indicate that VTE prophylaxis remains under-used in cancer patients (Streiff et al., 2023). Our study highlights the cost-effectiveness of DOACs in the primary prevention of VTE in cancer patients within the Chinese healthcare context. The comparative health benefits of DOACs, combined with reduced medical costs and the convenience of oral administration, make them a viable option for widespread clinical use, potentially leading to more efficient allocation of healthcare resources. In scenario analyses simulating real therapeutic settings, long-term prophylaxis and the use of generic DOACs were found to offer additional pharmacoeconomic advantages. Subgroup analyses revealed that apixaban, while slightly increasing both health benefits and medical costs, yielded an ICER below the willingness-to-pay threshold, indicating that apixaban is the preferred drug for VTE prevention among individual DOACs. These findings provide valuable information for the management and practice of VTE prophylaxis in clinical settings.

In base-case and PSA, DOACs have been demonstrated to be more cost-effective than LMWHs. However, the results were found to be sensitive to the relative risks of VTE and bleeding, which reflect the clinical benefits and harms associated with thromboprophylaxis in the cancer population. When poor protection or high bleeding risks were estimated in DOACs, the ICERs would become favorable for LMWHs. This indicates that the additional benefits of DOACs in primary thromboprophylaxis for cancer patients may be reduced in certain tumor types, such as gastrointestinal cancer, due to the higher associated risk of bleeding. Consistent with several clinical studies (Di Nisio et al., 2016; Agnelli, 2019), our research also observed a slight improvement in mortality with DOAC thromboprophylaxis compared to LMWH. However, the increased mortality highlights the pharmacoeconomic advantage of DOACs over LMWHs in primary thrombosis prevention. Therefore, it is recommended that the optimal thrombosis prevention strategy be tailored to the specific cancer type and stage. Moreover, drug–drug interactions (DDIs) can affect the safety and efficacy profiles of drug therapy. LMWHs have fewer DDIs due to their unique metabolic pathway and mechanism of action, whereas the liver metabolism of DOACs leads to more potential DDIs during their use (Tsoukalas et al., 2022). This could lead to intricate variations in the pharmacoeconomic findings of our study, which require further exploration.

The primary strength of this study lies in its comparison of the cost-effectiveness of available DOACs (rivaroxaban and apixaban) with LMWHs and no thromboprophylaxis in VTE prevention among the cancer population in China. However, there are several intrinsic limitations. First, a comprehensive analysis of 22 randomized controlled trials was conducted to assess the clinical outcomes of thromboprophylaxis on the prevention of cancer-associated VTE. Nevertheless, the inherent heterogeneity of different studies encompassing variations in patient cohorts, types of anticoagulants, dosages, and improvements in treatment regimens, nursing, and medical management over time may impact vital clinical outcomes and introduce bias into the final results. Second, the probabilities of clinical events were estimated based on the available RCTs with follow-up periods up to 1.5 years, which may introduce a potential bias when extrapolating these inputs to a longer time horizon. Third, it is important to note that the clinical outcomes obtained from the indirect network analysis, which included the Asia–Pacific cohort, may still be affected if real-world evidence based on the Chinese population is involved. This may result in systematic bias and limit the validity of our findings. Fourth, our model only considers the discontinuation of thromboprophylaxis following a major bleeding event. Other factors such as dosage variations, patient compliance, and switching between anticoagulants were not incorporated into this study due to the lack of specific data. Additionally, it is worth noting that LMWHs have fewer DDIs than DOACs. The disparity in DDIs may result in more intricate alterations in critical clinical outcomes, potentially compromising the validity of DOACs, particularly when dealing with populations taking multiple medications in real-world settings. Furthermore, DOACs’ benefits in primary thromboprophylaxis were currently obtained from three available RCTs, involving only apixaban and rivaroxaban. Variations in safety profiles in individual DOACs have been noticed (Attard et al., 2022; Ning et al., 2023), along with differences in bleeding risk based on cancer types (Farge et al., 2022). This highlights the importance of considering cancer types and updating of evidence of more individual anticoagulants when making the decision of thromboprophylaxis.

## 6 Conclusion

From the perspective of the Chinese payer, primary prophylaxis with DOACs is more cost-effective than LMWHs and no thromboprophylaxis, and apixaban was the preferred drug in VTE prevention. The results were sensitive to factors such as VTE and bleeding risk, the cost of anticoagulants, the self-payment ratio in Chinese Medicare, and the overall cancer death rate.

## Data Availability

The original contributions presented in the study are included in the article/Supplementary Material; further inquiries can be directed to the corresponding authors.
