# Supplementary material for: Cost-effectiveness analysis of direct oral anticoagulants versus low-molecular-weight heparin and no thromboprophylaxis in primary prevention of cancer-associated venous thromboembolism in China
Source: Front Pharmacol. 2024 Sep 23;15:1373333. doi: 10.3389/fphar.2024.1373333 (PMC11456474; doi:10.3389/fphar.2024.1373333)
Supplement: Supplementary file 1 [file DataSheet1.PDF]

SUPPLEMENTAL FILE

Title:Cost-effectiveness analysis of new oral anticoagulants versus low molecular weight heparin in primary thromboprophylaxis of venous thromboembolism in cancer patients in China.

Contents

Tables

Supplementary 1. Search strategy 2

Supplementary 2. Inclusion, exclusion criteria and study endpoints 4

Supplementary 3. Screening flow diagram of studies 6

Supplementary 4. Study characteristics 7

Supplementary 5. Bias risk assessments of included literatures 10

Supplementary 6. Funnel plot of network analysis 12

Supplementary 7. Funnel plot of probability of VTE (subgroup analysis) 13

Supplementary 8. Funnel plot of mortality (subgroup analysis) 14

Supplementary 9. Funnel plot of probability of bleeding (subgroup analysis) 15

Supplementary 10. Network analysis diagrams 16

Supplementary 11. Relative risk of VTE, bleeding and death obtained from network meta-analysis 17

Supplementary 12. Heterogeneity analysis in direct comparison 18

Supplementary 13 Incidence rare of VTE in Placebo 21

Supplementary 14 Incidence rare of bleeding in Placebo 22

Supplementary 15. Incremental CE Scatter Plot of individual DOACs and LMWHs vs Placebo for 5 years 23

Supplementary 16. Incremental CE Scatter Plot of individual DOACs and LMWHs vs Placebo for 10 years 24

Supplementary 17. Incremental CE Scatter Plot of generic drugs comparison 25

Supplementary 1. Search strategy

(1) Web of science

#1	TS= (New oral anticoagulant*) OR AB=(DOAC) OR AB=(NOAC) OR AB=(acting oral anticoagulant*) OR AB=(non-vitamin K antagonist oral) OR AB=(Anticoagulant*) OR AB=(direct oral anticoagulants*) OR AB=(new oral anticoagulant*) OR AB=(novel oral anticoagulant*) OR AB=(rivaroxaban) OR AB=(Dabigatran) OR AB=(Apixaban) OR AB=(Edoxaban)
#2	TS= (low-molecular-weight heparin*) OR AB=(LMWH) OR AB=(Nadroparin) OR AB=(Dalteparin*) OR AB=(Certoparin*) OR AB=(Enoxaparin) OR AB=(Tinzaparin*) OR AB=(Bemiparin*) OR AB=(Anticoagulant*)
#3	TS= (venous thromboembolism*) OR AB=(venous thromboembolism*) OR AB=(venous thrombosis*) OR AB=(deep vein thrombosis*) OR AB=(pulmonary embolism) OR AB=(lung embolism) OR AB=(DVT) OR AB=(VTE) OR AB=(PE)
#4	TS= (malignan*) OR AB=(neoplasm*) OR AB=(cancer*) OR AB=(carcinoma*) OR AB=(adenocarcinoma*) OR AB=(tumour*)
#5	TI= (review) OR DT=(Review) OR TI=(guideline*) OR TI=(CASE PRESENTATION) OR TI=(case report*) OR TI=(case of)
#6	#1 OR #2
#7	#3 AND #4 AND #6
#8	(#7) NOT #5
#9	ALL= (clinical trial) OR ALL=(Intervention Study) OR ALL=(randomized controlled trial) OR ALL=(controlled clinical trial) OR ALL=(clinical observation)
#10	#8 AND #9

(2) Pubmed

#1	((((((((((((((New oral anticoagulant*[MeSH Terms]) OR (novel oral anticoagulant*[MeSH Terms])) OR (Direct Oral Anticoagulant*[MeSH Terms])) OR (Non-vitamin antagonist oral anticoagulant*[MeSH Terms])) OR (New oral anticoagulant*[Title/Abstract])) OR (novel oral anticoagulant*[Title/Abstract])) OR (Direct Oral Anticoagulant*[Title/Abstract])) OR (Non-vitamin antagonist oral anticoagulant*[Title/Abstract])) OR (Factor Xa Inhibitor*[Title/Abstract])) OR (DOAC[Title/Abstract])) OR (NOAC[Title/Abstract])) OR (rivaroxaban[Title/Abstract])) OR (Dabigatran[Title/Abstract])) OR (Apixaban[Title/Abstract])) OR (Edoxaban[Title/Abstract]))
#2	((((((((((low-molecular-weight heparin[MeSH Terms]) OR (LMWH[MeSH Terms])) OR (low-molecular-weight heparin[Title/Abstract])) OR (LMWH[Title/Abstract])) OR (anticoagulant[MeSH Terms])) OR (Nadroparin[Title/Abstract])) OR (Dalteparin[Title/Abstract])) OR (Certoparin[Title/Abstract])) OR (Enoxaparin[Title/Abstract])) OR (Tinzaparin[Title/Abstract])) OR (Bemiparin[Title/Abstract]))
#3	((((((((venous thromboembolism*[MeSH Terms]) OR (venous thromboembolism*[Title/Abstract])) OR (venous thrombosis*[Title/Abstract])) OR (deep vein thrombosis*[Title/Abstract])) OR (pulmonary embolism*[Title/Abstract])) OR (lung embolism*[Title/Abstract])) OR (DVT[Title/Abstract])) OR (VTE[Title/Abstract])) OR (PE[Title/Abstract]))
#4	(((((((malignan*[MeSH Terms]) OR (malignan*[Title/Abstract])) OR (neoplasm*[Title/Abstract])) OR (cancer*[Title/Abstract])) OR (carcinoma*[Title/Abstract])) OR (adenocarcinoma*[Title/Abstract])) OR (tumour*[Title/Abstract]))
#5	((((((clinical trial[MeSH Terms]) OR (clinical trial[Title/Abstract])) OR (Intervention Study[Title/Abstract])) OR (randomized controlled trial[Title/Abstract])) OR (controlled clinical trial[Title/Abstract])) OR (clinical observation[Title/Abstract]))
#6	((((((review[Title]) OR (review[Publication Type])) OR (guideline*[Title])) OR (CASE PRESENTATION[Title/Abstract])) OR (case report*[Title])) OR (case of[Title]))
#7	#1 OR #2 AND #3 AND #4 AND #5
#8	#7 NOT #6

（3）Cnki.net

#1	(Subject: New oral anticoagulant + Direct Oral Anticoagulant) or (Abstract: New oral anticoagulant + Factor Xa Inhibitor + Direct Oral Anticoagulant + Rivaroxaban + Dabigatran + Apixaban + Edoxaban)
#2	(Subject: low-molecular-weight heparin) or (Abstract: low-molecular-weight heparin + Nadroparin + Dalteparin + Certoparin + Enoxaparin +Tinzaparin + Bemiparin)
#3	(Subject: venous thromboembolism) or (Abstract: venous thromboembolism + deep vein thrombosis + pulmonary embolism + lung embolism)
#4	(Subject: tumor) or (Abstract: malignancy + neoplasm + cancer + tumor)
#5	(Subject: clinical trial + Intervention Study + randomized controlled trial + controlled clinical trial + clinical observation)
#6	(Subject: review + guideline + case presentation)
#7	#1 OR #2 AND #3 AND #4 AND #5
#8	#7 NOT #6

Supplementary 2. Inclusion, exclusion criteria and study endpoints

Inclusion criteria: This study focuses on randomized controlled trials (RCTs) comparing DOACs and LMWHs for the prevention of cancer-associated venous thromboembolism (CAVTE), with no language restrictions. The study subjects are cancer patients who are receiving non-surgical treatment regimens and have not yet developed VTE, with no limitations on cancer types. The interventions include the use of DOACs (apixaban, rivaroxaban, edoxaban, dabigatran) or LMWHs (nadroparin, dalteparin, certoparin, enoxaparin, tinzaparin, bemiparin, calcium heparin, etc.) for prevention, with control measures including the use of a placebo or no anticoagulant. The outcome measures are: incidence of first VTE (including any venous thromboembolism, deep vein thrombosis, pulmonary embolism, lung embolism), all-cause mortality, and major bleeding events (including gastrointestinal hemorrhage, intracranial hemorrhage and clinic relevant non-major bleeding).

Exclusion criteria: The literature type was limited to original research articles; Experimental designs or outcomes that did not meet the requirements were excluded; Participants with a confirmed history of VTE or central venous catheterization were excluded; Studies conducted in the perioperative period were excluded; Research focusing on the treatment of tumor-associated thrombosis was also excluded. In cases where duplicate data was found, only the study with the most complete data was retained.

Supplementary 3. Screening flow diagram of studies

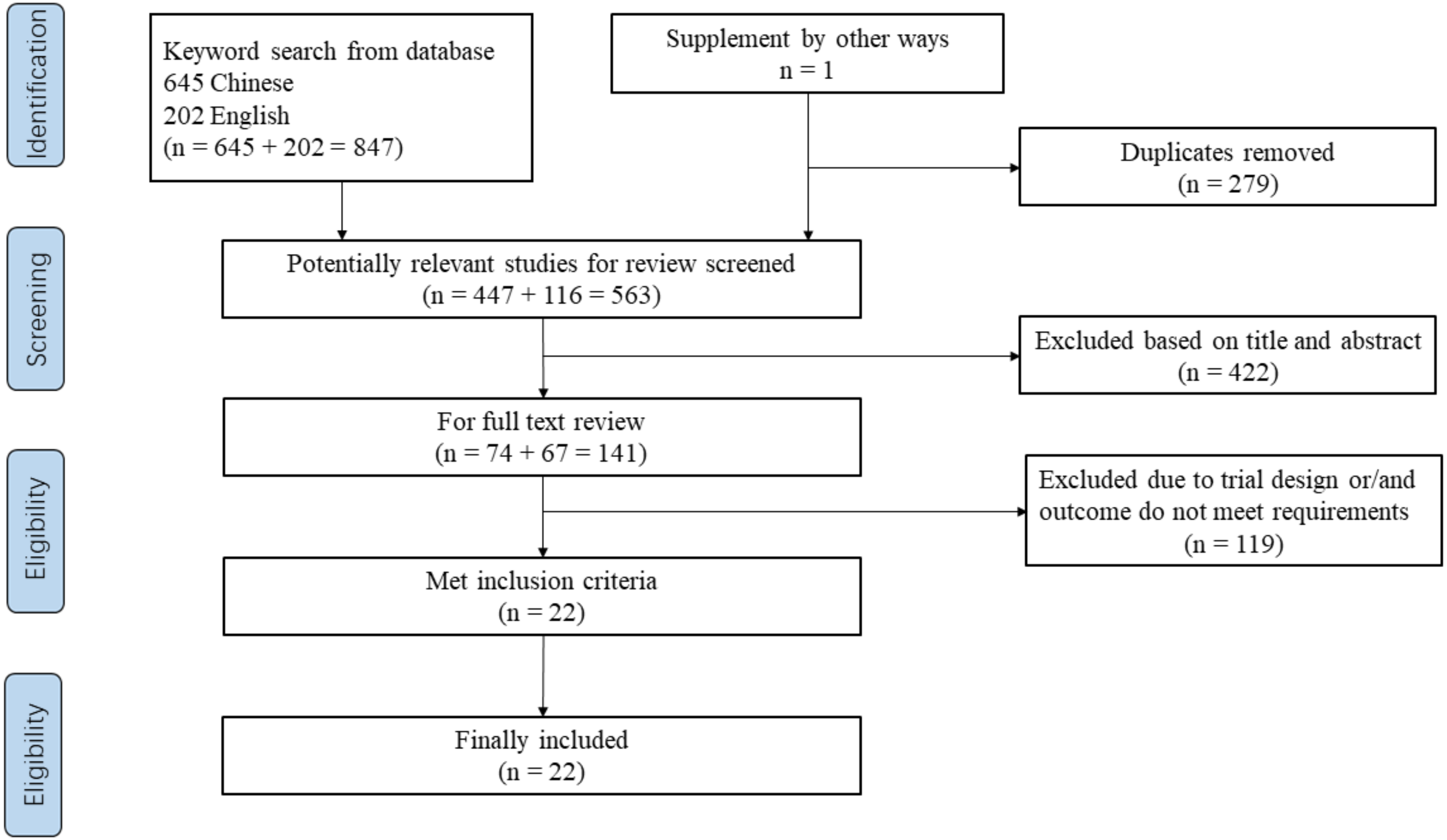

Supplementary 4. Study characteristics

Study	Cases		Mean age		Type of cancer	Treatment		Outcomes	Median follow-up
	Treat	Control	Treat	Control		Treat	Control		
Agnelli 2009	769	381	62	64	Metastatic or locally advanced lung, gastrointestinal (stomach, colon, or rectum), pancreatic, breast, ovarian, or head and neck cancer	Nadroparin	Placebo	VTE, DVT, PE, AE, bleeding, major bleeding minor bleeding, death	112 days
Altinbas 2004	42	42	58	58	Small cell lung cancer	Dalteparin	No drug	DVT, death	10 months
Haas 2012	442	441	55	57	Disseminated metastatic breast carcinoma or non-small cell lung carcinoma of stage III or IV	Certoparin	Placebo	VTE, DVT, PE, bleeding, major bleeding, minor bleeding	6 months
Kakkar 2004	190	184	62	61	stage III or IV (locally advanced or metastatic) malignant cancer of the breast, lung, gastrointestinal tract, pancreas, liver, genitourinary tract, ovary, or uterus	Dalteparin	Placebo	DVT, PE, bleeding, major bleeding, minor bleeding, death	6 months
Khorana 2017	50	48	60	58	Malignant tumors	Dalteparin	No drug	VTE,DVT,PE, bleeding, major bleeding, minor bleeding	12 weeks
Klerk 2005	148	154	63	64	Histologically documented solid malignant tumors	LMWH	Placebo	VTE, bleeding, major bleeding, minor bleeding	1 year
Lecumberri 2013	20	18	61	64	Small cell lung cancer	Bemiparin	No drug	bleeding, major bleeding, minor bleeding, death	6 months
Macbeth 2016	1101	1101	65	64	Lung cancer	Dalteparin	No drug	VTE,DVT,PE, AE, bleeding, major bleeding	23 months
Meyer 2018	269	280	62	62	Stage I, II or IIIA Non-small cell lung cancer	Tinzaparin	No drug	VTE, bleeding, major bleeding, minor bleeding	12 months

Pelzer 2015	160	152	62	63	Pancreatic adenocarcinoma	Enoxaparin	No drug	DVT,PE, major bleeding	3 months
Perry 2010	99	87	57	55	Grade 3 or grade 4 glioma (anaplastic astrocytoma, glioblastoma multiforme, gliosarcoma, anaplastic oligodendroglioma or anaplastic mixed glioma)	Dalteparin	Placebo	DVT,PE, major bleeding, death	6 months
Sideras 2006	68	70	70	64	Malignant tumors	Dalteparin	No drug	minor bleeding, death	18 months
Saroj Vadhan-Raj 2013	23	11	63	63	Advanced Pancreatic Cancer	Dalteparin	No drug	VTE, DVT, PE	6 months
Van Doormaal 2011	244	259	65	65	Non-small-cell lung cancer	Nadroparin	No drug	VTE, DVT,PE, AE, bleeding, major bleeding, minor bleeding, death	10 months
Zhang 2013	44	41	56	56	Lung cancer	Nadroparin	No drug	DVT, PE, bleeding, major bleeding, minor bleeding,	3 months
Zhu 2014	23	23	55	56	Stomach cancer	Dalteparin	No drug	DVT	2 months
Zwicker 2013	23	11	65	64	Malignant tumors	Enoxaparin	No drug	VTE, death	2 months
Wang 2005	27	32	59	59	Non-small-cell lung cancer	LMWH	No drug	PE, VTE, bleeding, death	Uninformed
EK 2018	186	191	67	68	Small cell lung cancer	Enoxaparin	No drug	VTE, major bleeding, PE, death	41 months
Carrier 2019	288	275	61	61	Primary cancer (brain, bladder ,lung, testicular, stomach ,pancreatic ,lymphoma, myeloma, gynecologic, colon,	Apixaban	Placebo	VTE, DVT, PE, bleeding, major bleeding, CRNMB, death	183days

					prostate)				
Khorana 2019	420	421	63	62	Primary cancer(pancreatic ,breast, gastric, gastroesophageal junctional, genitourinary ,lung, lymphoma, ovarian)	Rivaroxaban	Placebo	VTE, DVT, PE, bleeding, major bleeding, CRNMB, death	180days
Levine 2017	95	30	60	59	Advanced or metastatic lung, breast, GI (colon, rectum, pancreas, stomach), bladder, cancer of unknown origin, ovarian or prostate cancer, myeloma or selected lymphomas	Apixaban	Placebo	VTE, DVT, PE, bleeding, major bleeding, CRNMB, death	84days

Supplementary 5. Bias risk assessments of included literatures

Cochrane bias risk assessments were conducted using RevMan 5.3 software. All 22 included studies were randomized controlled trials. The randomization methods mentioned in these studies mostly involved computer-generated randomization or the envelope method. Nine studies explicitly reported using double-blind methods, resulting in objective outcome data and were therefore assessed as low risk. Five studies mentioned open-label designs, and were thus assessed as high risk for implementation bias. None of the studies reported other sources of bias, and any unmentioned aspects were rated as uncertain

5.1 Risk of bias graph: percentages

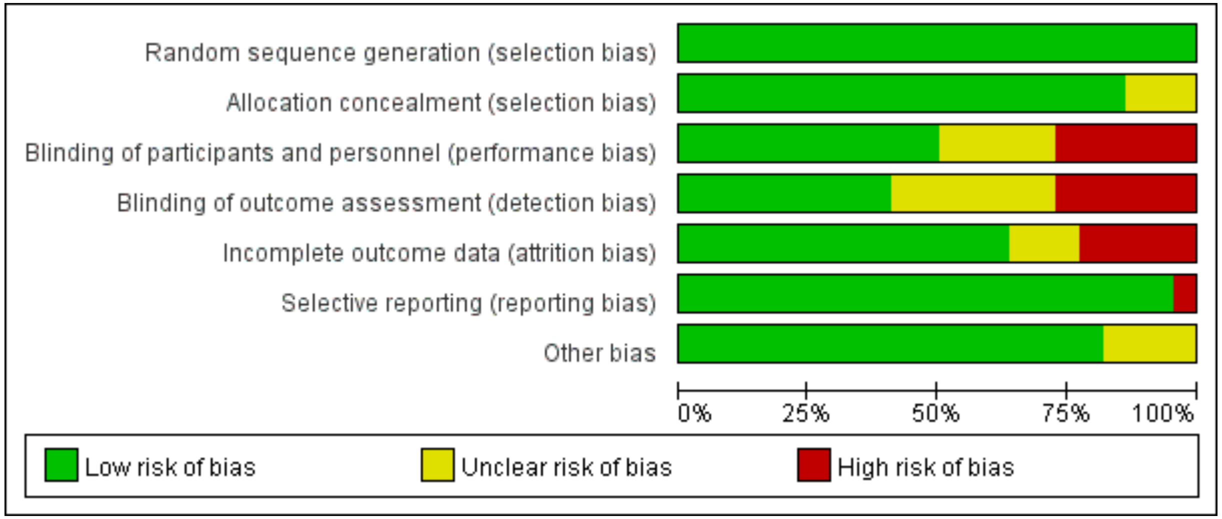

5.2 Risk of bias summary

	Random sequence generation (selection bias)	Allocation concealment (selection bias)	Blinding of participants and personnel (performance bias)	Blinding of outcome assessment (detection bias)	Incomplete outcome data (attrition bias)	Selective reporting (reporting bias)	Other bias
Agnelli 2009	+	+	+	+	?	+	+
Altinbas 2004	+	?	−	−	?	+	+
Carrier 2019	+	+	+	+	−	+	+
EK 2018	+	+	−	−	−	+	+
Haas 2012	+	+	+	+	?	+	+
Kakkar 2004	+	+	+	+	−	+	?
Khorana 2017	+	+	?	?	+	+	+
Khorana 2019	+	+	+	+	+	+	+
Klerk 2005	+	+	+	?	+	+	+
Lecumberri 2013	+	+	−	−	−	+	?
Levine 2012	+	+	+	+	−	+	+
Macbeth 2016	+	+	−	−	+	+	+
Meyer 2018	+	+	−	−	+	+	+
Pelzer 2015	+	+	−	−	+	+	?
Perry 2010	+	+	+	+	+	+	+
Sideras 2006	+	+	+	+	+	+	+
Vadhan-Raj 2013	+	+	?	?	+	+	?
Van Doormaal 2011	+	+	+	?	+	+	+
Wang 2005	+	+	?	?	+	+	+
Zhang 2013	+	?	?	?	+	+	+
Zhu 2014	+	?	?	?	+	+	+
Zwicker 2013	+	+	+	+	+	−	+

Supplementary 6. Funnel plot of network analysis

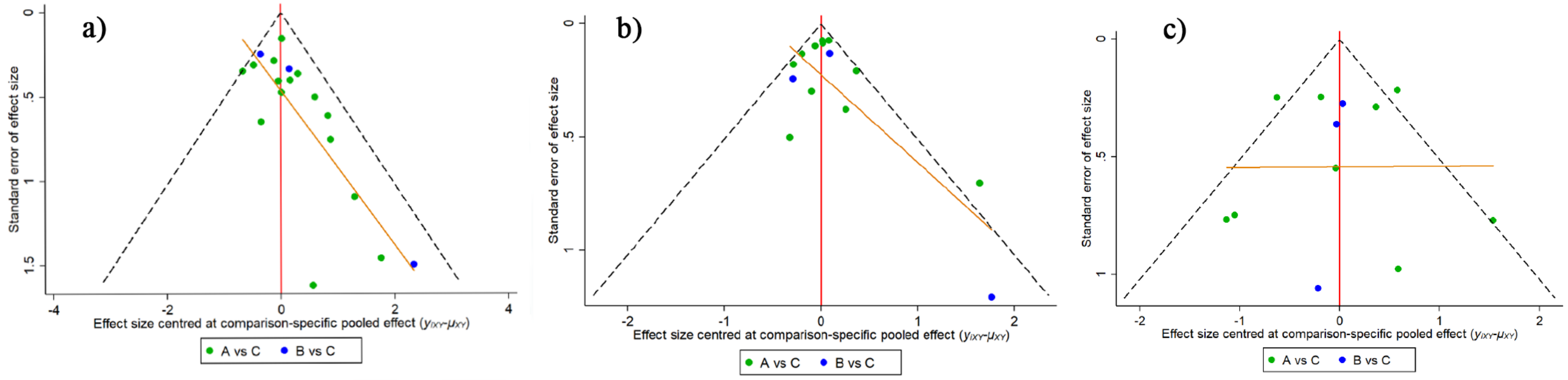

Funnel plot of network analysis for a) VTE; b) death; c) bleeding, Durg: (A)LMWHs; (B)DOACs; (C)Placebo

Supplementary 7. Funnel plot of probability of VTE (subgroup analysis)

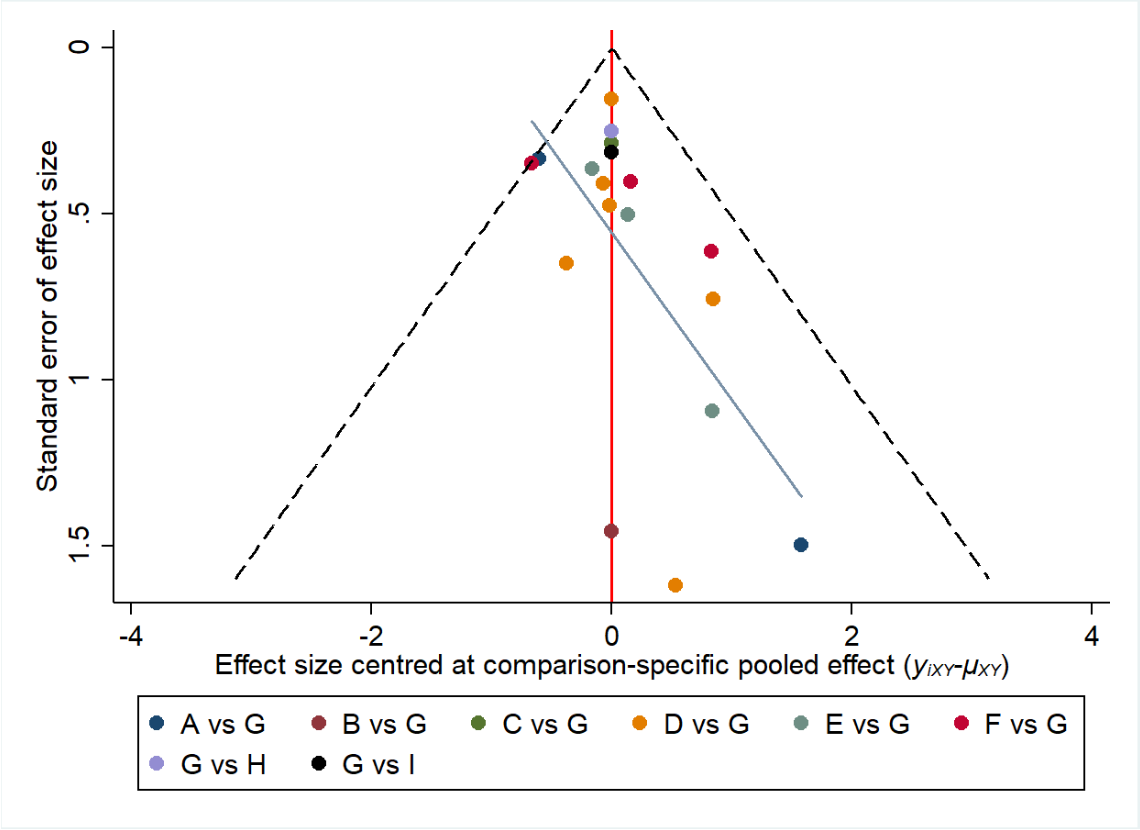

(A)Apixaban; (B)Bemiparin; (C)Certoparin; (D)Dalteparin; (E)Enoxaparin; (F)Nadroparin; (G)Placebo; (H)Rivaroxaban; (I)Tinzaparin

Supplementary 8. Funnel plot of mortality (subgroup analysis)

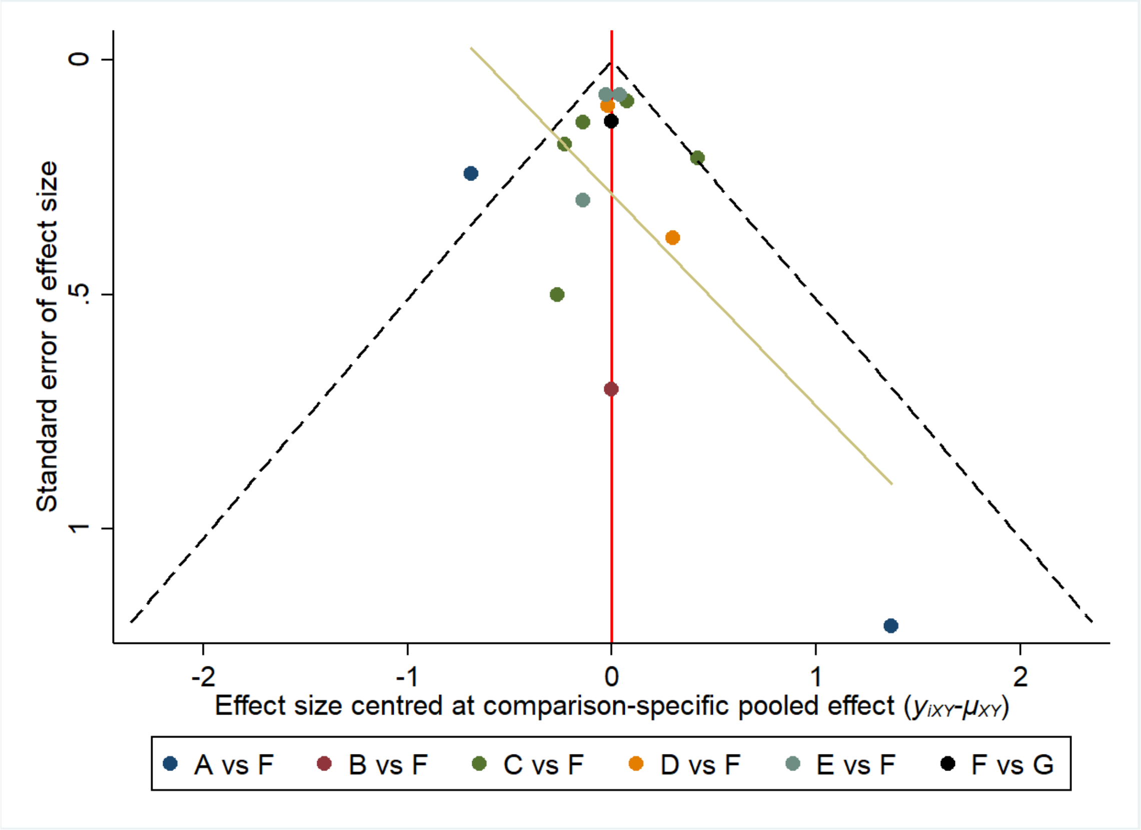

(A)Apixaban; (B)Bemiparin; (C)Dalteparin; (D)Enoxaparin; (E)Nadroparin; (F)Placebo; (G)Rivaroxaban

Supplementary 9. Funnel plot of probability of bleeding (subgroup analysis)

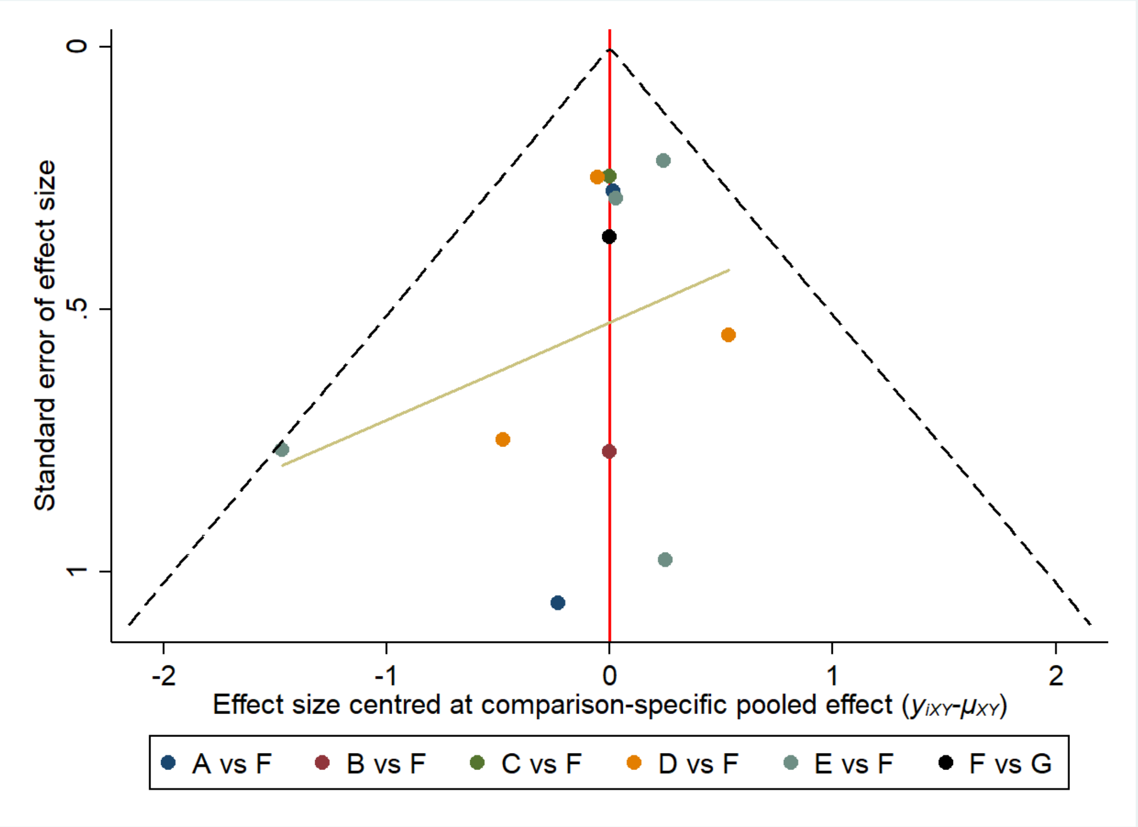

(A)Apixaban; (B)Bemiparin; (C)Certoparin; (D)Dalteparin; (E)Nadroparin; (F)Placebo; (G)Rivaroxaban

Supplementary 10. Network analysis diagrams

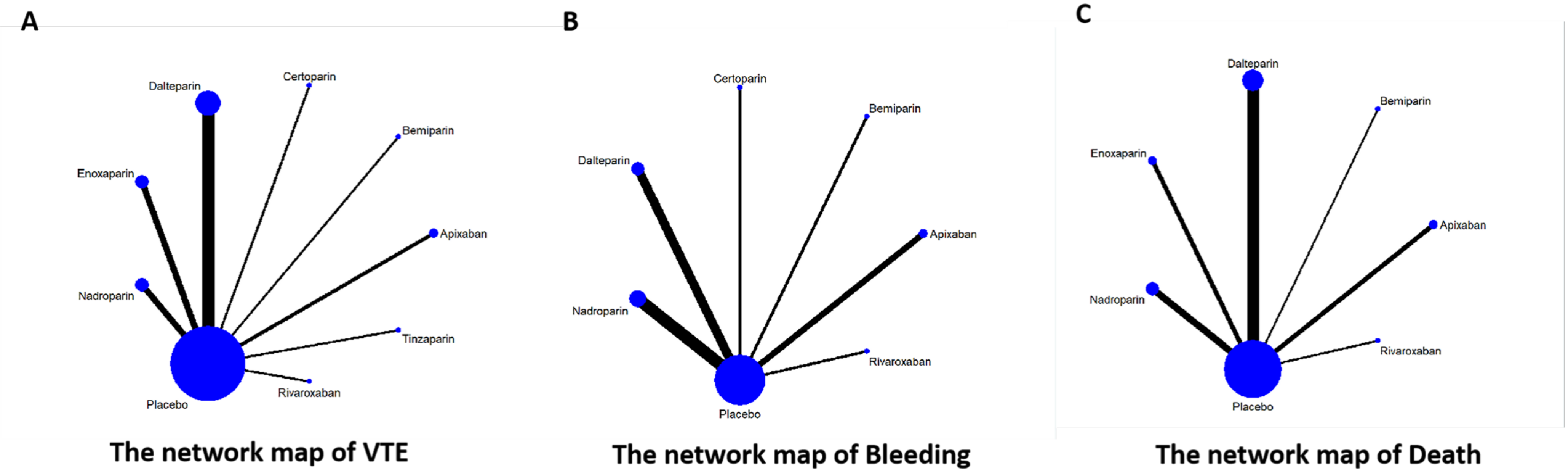

Open-loop diagrams were obtained through network analysis, indirectly comparing DOACs with LMWHs and no thromboprophylaxis. The size of the nodes in the diagrams indicates that the no-anticoagulation group had the largest patient sample size, while the width of the lines shows that the greatest number of studies were conducted on dalteparin.

Supplementary 11. Relative risk of VTE, bleeding and death obtained from network meta-analysis

Compared with placebo, DOACs, including individual apixaban and rivaroxaban, reduce the risk of VTE without increasing the risks of bleeding and death.

Subgroup	n	No.of event	Relative risk(95% CI)
**VTE**			
Placebo	3885	344	
NOACs	803	37	0.54(0.37-0.80)
LMWHs	3555	166	0.58(0.48-0.71)
Apixaban	383	12	0.37(0.19-0.70)
Rivaroxaban	420	25	0.68(0.42-1.10)
**Bleeding**			
Placebo	3346	144	
NOACs	788	56	1.56(0.78-3.11)
LMWHs	3013	225	1.67(1.12-2.50)
Apixaban	383	37	1.55(0.59-4.08)
Rivaroxaban	405	19	1.58(0.49-5.08)
**Death**			
Placebo	2171	759	
NOACs	803	120	0.90(0.72-1.13)
LMWHs	1839	597	0.92(0.86-0.99)
Apixaban	383	36	1.14(0.72-1.82)
Rivaroxaban	420	84	0.84(0.65-1.09)

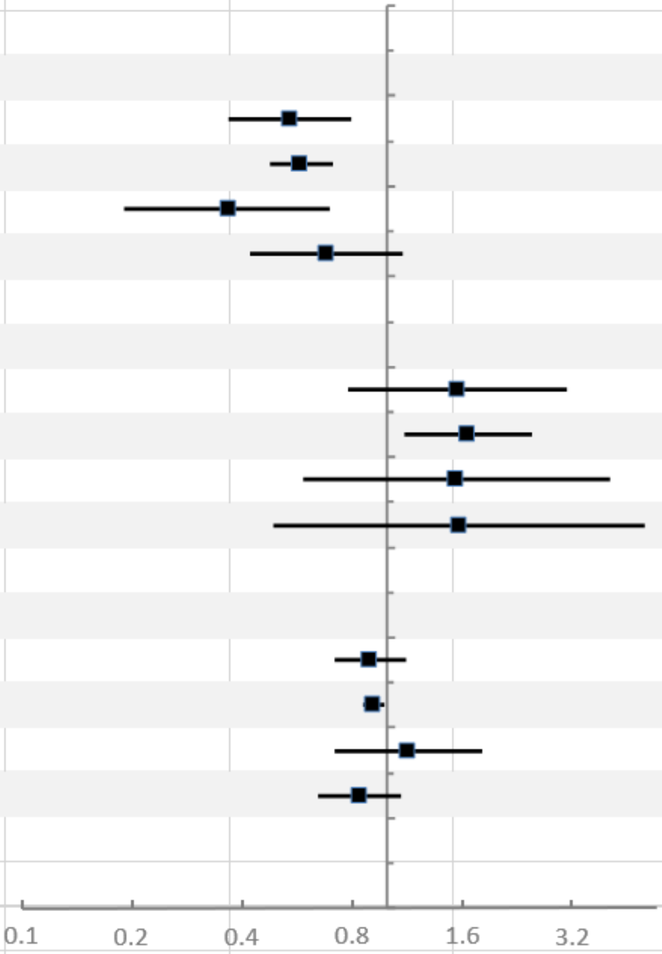

Supplementary 12. Heterogeneity analysis in direct comparison

Relative risk of VTE in DOACs v. Placebo							
study	year	evente	ne	eventc	nc	P-value	I^2
Carrier	2019	12	288	28	275	0.120	52.9%
Khorana	2019	25	420	37	421		
Levine	2012	0	95	3	30		
Relative risk of death in DOACs v. Placebo							
study	year	evente	ne	eventc	nc	P-value	I^2
Carrier	2019	35	288	27	275	0.130	50.9%
Khorana	2019	84	420	100	421		
Levine	2012	1	95	2	30		
Relative risk of bleeding in DOACs v. Placebo							
study	year	evente	ne	eventc	nc	P-value	I^2
Carrier	2019	31	288	20	275	0.969	0.0%
Khorana	2019	19	405	12	404		
Levine	2012	6	95	1	30		
Relative risk of VTE in LMWHs v. Placebo							
study	year	evente	ne	eventc	nc	P-value	I^2
Agnelli	2009	12	769	12	381	0.389	5.6%
Altinbas	2004	0	42	1	42		
Haas	2012	19	442	29	441		
Khorana	2017	6	50	10	48		
Lecumberri	2013	0	20	4	18		
Macbeth	2016	61	1101	107	1101		
Meyer	2018	18	269	20	280		
Pelzer	2015	10	160	22	152		
Perry	2010	9	99	13	87		
Sideras	2006	4	68	5	70		
Saroj Vadhan-Raj	2013	2	38	8	37		

Van Doormaal	2011	16	244	15	259		
Zhang	2013	3	44	11	41		
Zwicker	2013	1	23	3	11		
Relative risk of death in LMWHs v. Placebo							
study	year	evente	ne	eventc	nc	P-value	I^2
Agnelli	2009	33	769	16	381	0.101	37.3%
Altinbas	2004	18	42	28	42		
Kakkar	2004	105	190	112	184		
Khorana	2017	8	50	6	48		
Klerk	2005	97	148	118	154		
Lecumberri	2013	2	20	10	18		
Perry	2010	45	99	32	87		
Sideras	2006	45	68	41	70		
Van Doormaal	2011	138	244	160	259		
Zwicker	2013	9	23	6	11		
EK	2018	97	186	101	191		
Relative risk of bleeding in LMWHs v. Placebo							
study	year	evente	ne	eventc	nc	P-value	I^2
Agnelli	2009	57	769	30	381	0.002	67.2%
Haas	2012	46	447	23	451		
Kakkar	2004	9	190	5	184		
Khorana	2017	10	50	2	48		
Klerk	2005	10	148	2	154		
Lecumberri	2013	2	20	5	18		
Macbeth	2016	66	1101	21	1101		
Van Doormaal	2011	23	244	21	259		
Zhang	2013	2	44	2	41		

evente: number of events occurring in the experimental group; ne: total number of experimental group; event: number of events occurring in control group, nc: total number of experimental group.

I^2 and P-value are a measure of heterogeneity. P>0.1 indicates no heterogeneity, P<0.1 indicates the presence of heterogeneity. I^2 0-25%: no heterogeneity ; I^2 25-50%: mild heterogeneity ;

I^2 50-75%: moderate heterogeneity ; I^2 75-100%: high heterogeneity.

Supplementary 13 Incidence rare of VTE in Placebo

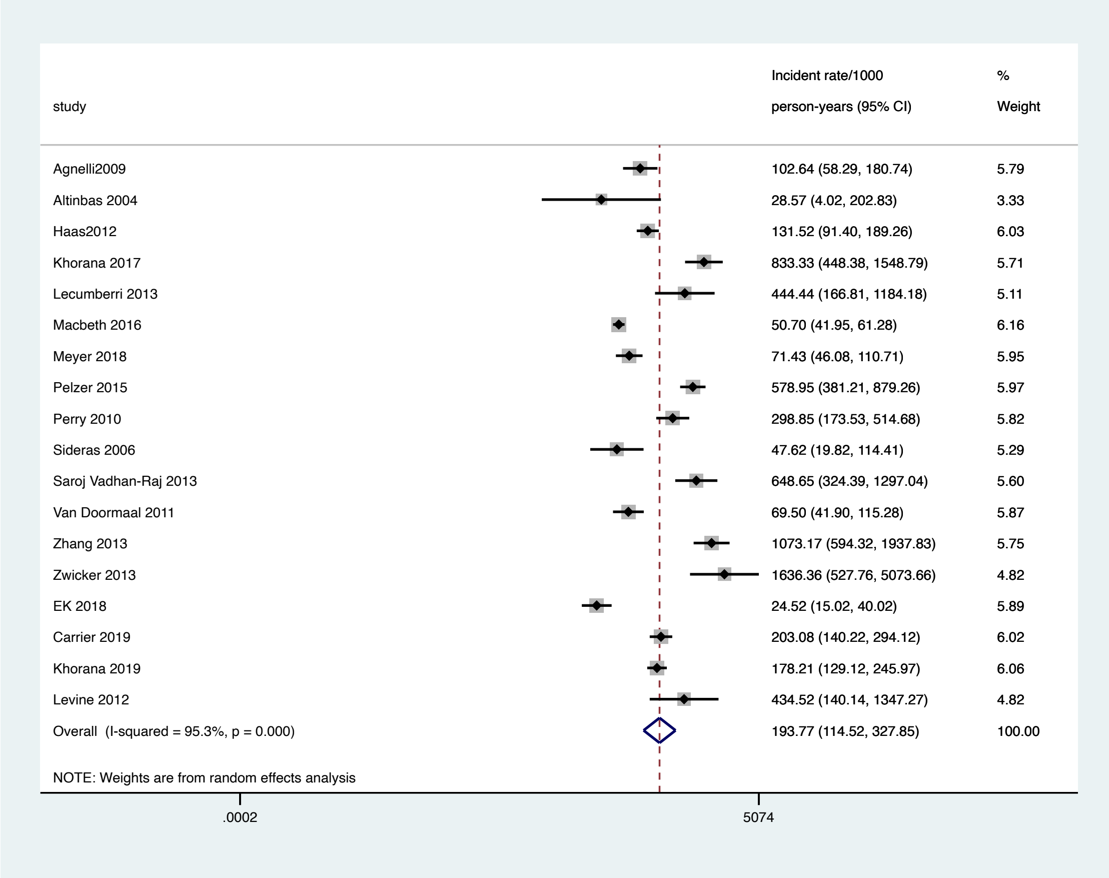

Heterogeneity chi-squared=360.57(d.f.=17) p=0.000； I-squared (variation in Es attributable to heterogeneity)=95.3%； Estimate of between-study variance Tau-squared= 1.1602

Supplementary 14 Incidence rare of bleeding in Placebo

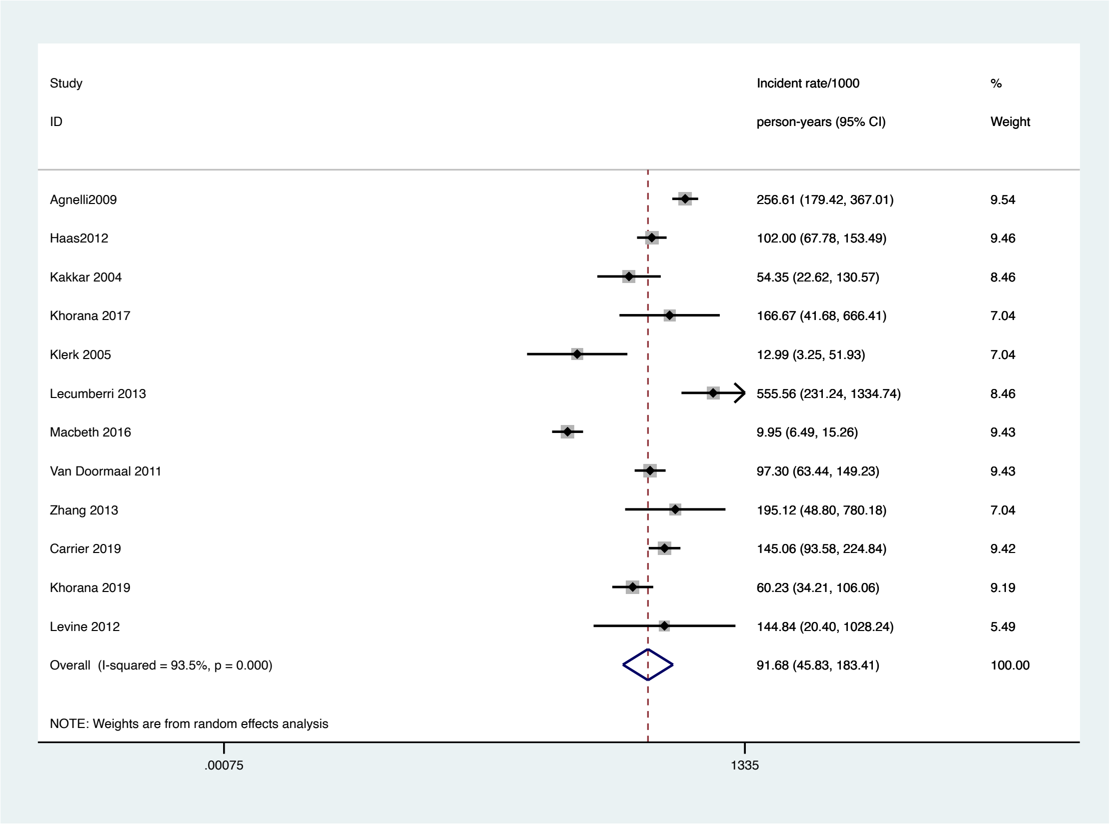

Heterogeneity chi-squared=169.26(d.f.=11)p=0.000；I-squared (variation in ES attributable to heterogeneity)=93.5%；Estimate of between-study variance Tau-squared= 1.2790；

Supplementary 15. Incremental CE Scatter Plot of individual DOACs and LMWHs vs Placebo for 5 years

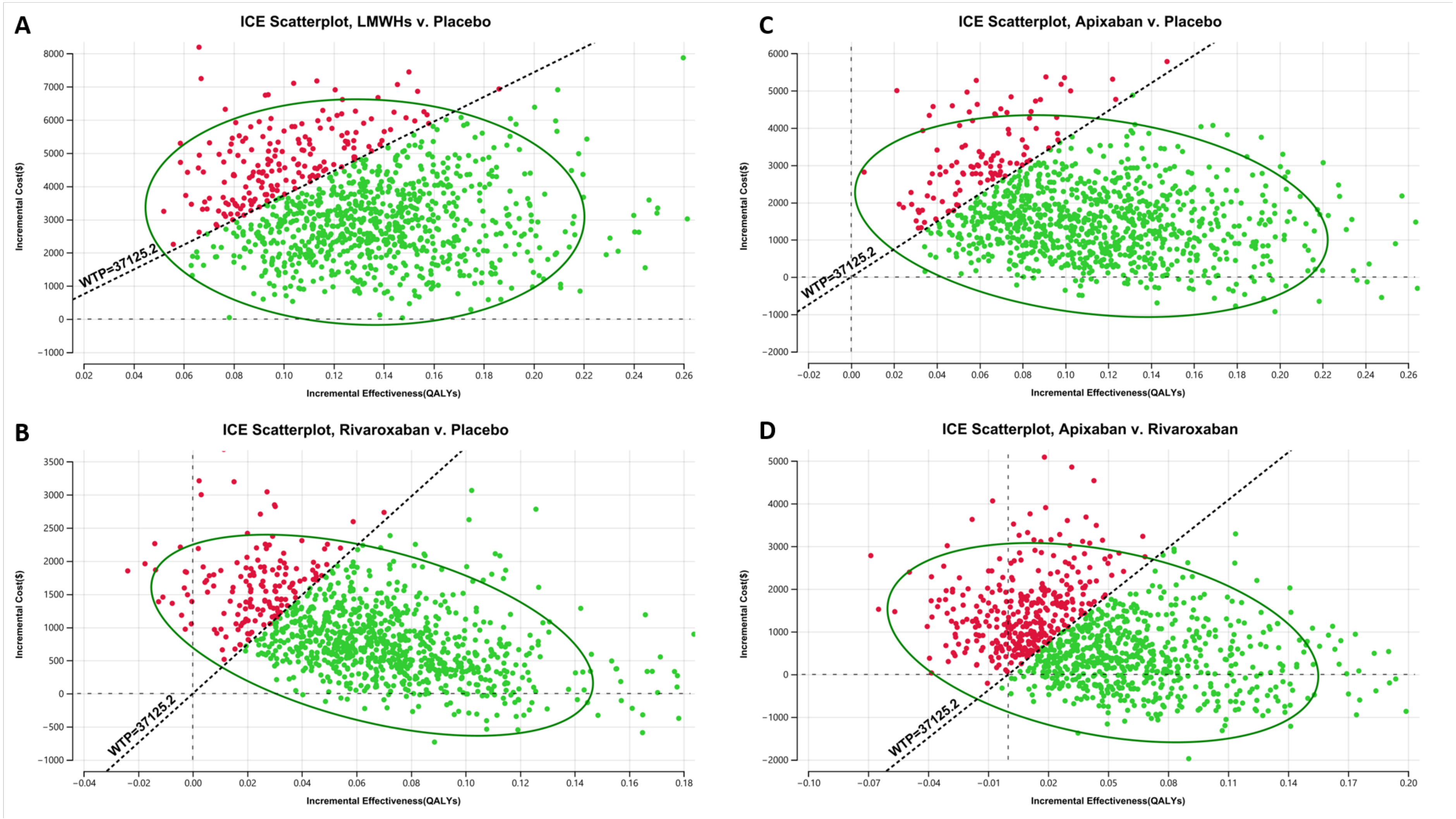

Scatter plot diagram illustrated the results of a 10,000-subject Monte Carlo simulation, A) LMWHs vs Placebo; B) Rivaroxaban vs Placebo; C) Apixaban vs Placebo; D) Aixaban vs Rivaroxaban.

Supplementary 16. Incremental CE Scatter Plot of individual DOACs and LMWHs vs Placebo for 10 years

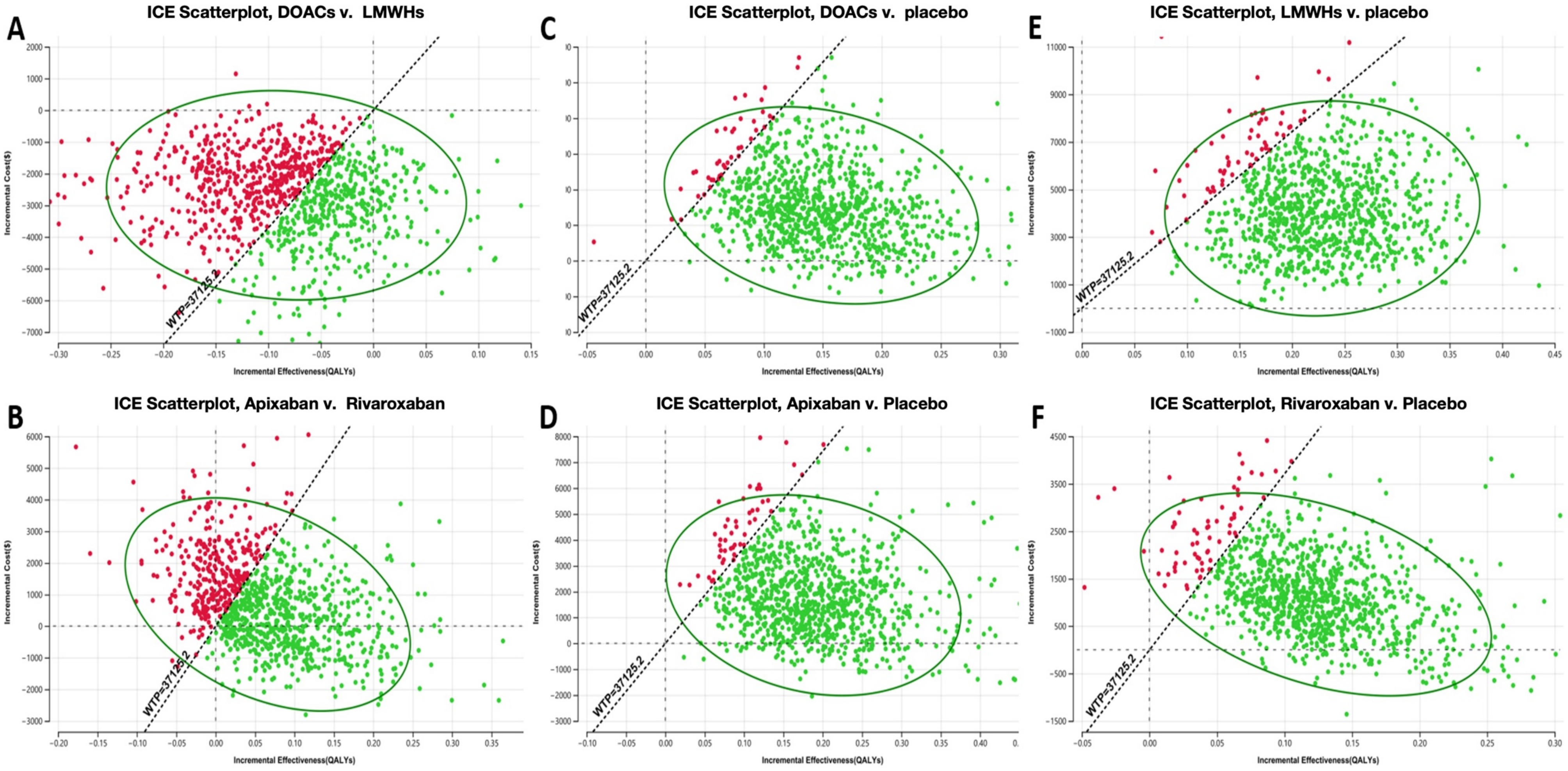

Scatter plot diagram illustrated the results of a 10,000-subject Monte Carlo simulation, A) DOACs vs LMWHs; B) Apixaban vs Rivaroxaban; C) DOACs vs Placebo; D) Aixaban vs Placebo; E) LMWHs vs Placebo; E) Rivaroxaban vs Placebo.

Supplementary 17. Incremental CE Scatter Plot of generic drugs comparison

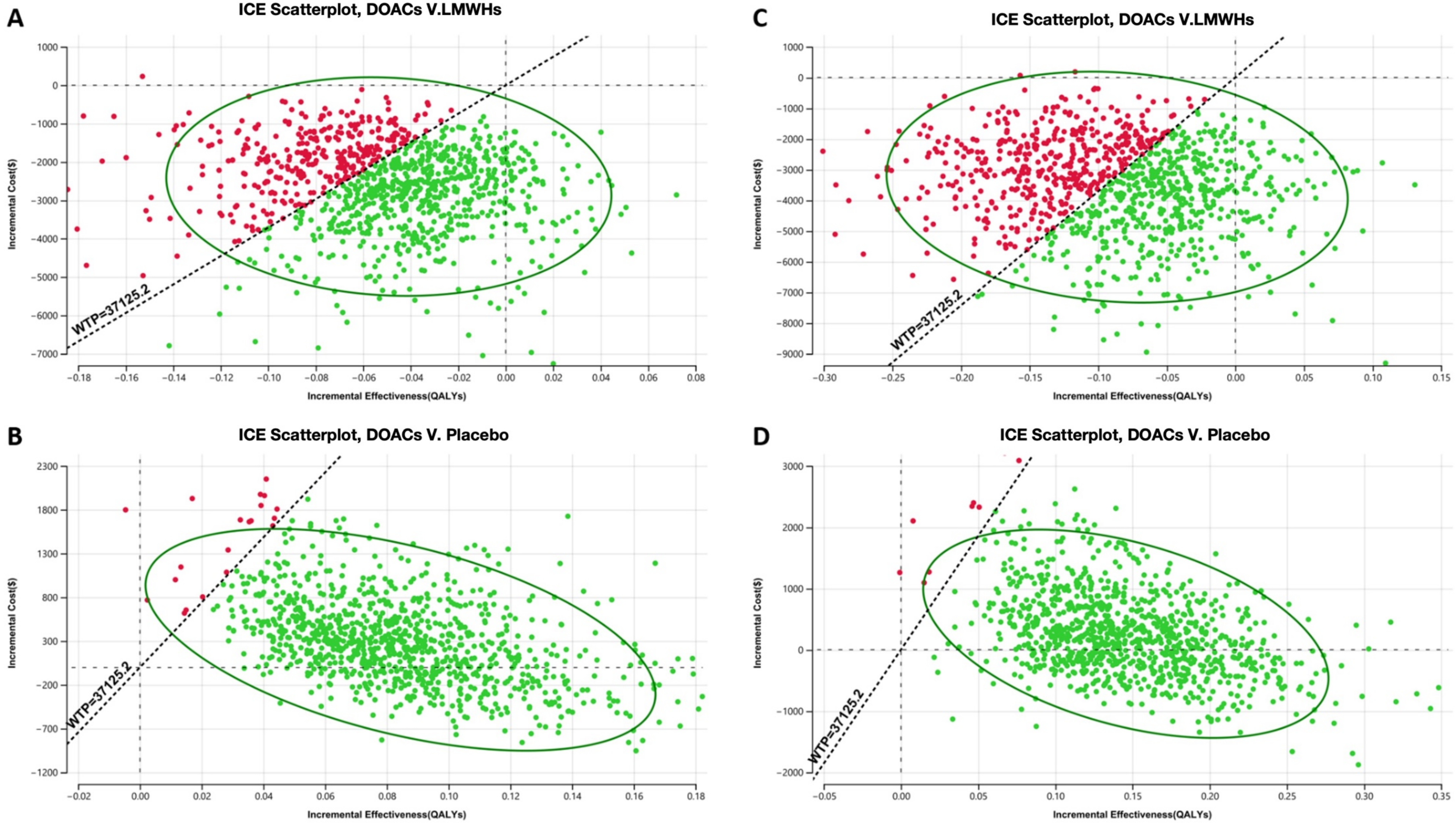

Scatter plot diagram illustrated the results of a 10,000-subject Monte Carlo simulation, (A) generic DOACs vs generic LMWHs for a 5 years horizon;(B) generic DOACs vs generic placebo for a 5 years horizon;(C) generic DOACs vs generic LMWHs for a 10 years horizon;(D) generic DOACs vs generic placebo for a 10 years horizon.
